# Supplementary material for: Identifying the impact of the covalent-bonded carbon matrix to FeN4 sites for acidic oxygen reduction
Source: Nat Commun. 2022 Jan 10;13:57. doi: 10.1038/s41467-021-27735-1 (PMC8748808; doi:10.1038/s41467-021-27735-1)
Supplement: Supplementary file 1 — Supplementary Information PDF [file 41467_2021_27735_MOESM1_ESM.pdf]

Identifying the impact of the covalent-bonded carbon matrix to FeN_4 sites for acidic oxygen reduction

Xueli Li, Zhonghua Xiang*

State Key Laboratory of Organic-Inorganic Composites, College of Chemical Engineering, Beijing University of Chemical Technology, Beijing, 100029, PR China

*Corresponding author. Email: xiangzh@mail.buct.edu.cn (Z. X.)

Chemicals. Ferric chloride [$FeCl_3$, Meryer, CAS#: 7705-08-0], Tetracyanoethylene [C6N4, Macklin, CAS#: 670-54-2], pyromellitic dianhydride (PMDA) [C10H2O6, aladdin, CAS#: 89-32-7], 1,4,5,8-Naphthalenetetracarboxylic dianhydride [C14H4O6, Macklin, CAS#: 81-30-1], 3,4,9,10-Perylenetetracarboxylic dianhydride [C24H8O6, Macklin, CAS#: 128-69-8], urea [$(NH_2)_2CO$, Heowns, CAS#: 57-13-6], ammonium chloride [NH_4Cl, Macklin, CAS#: 12125-02-9], ammonium molybdate tetrahydrate [$(NH_4)_6Mo_7O_{24}\cdot 4H_2O$, Macklin, CAS#: 12054-85-2], cabot Vulcan XC-72 [C, Macklin, CAS#: 1333-86-4] were used without further purification.

Supplementary Figures:

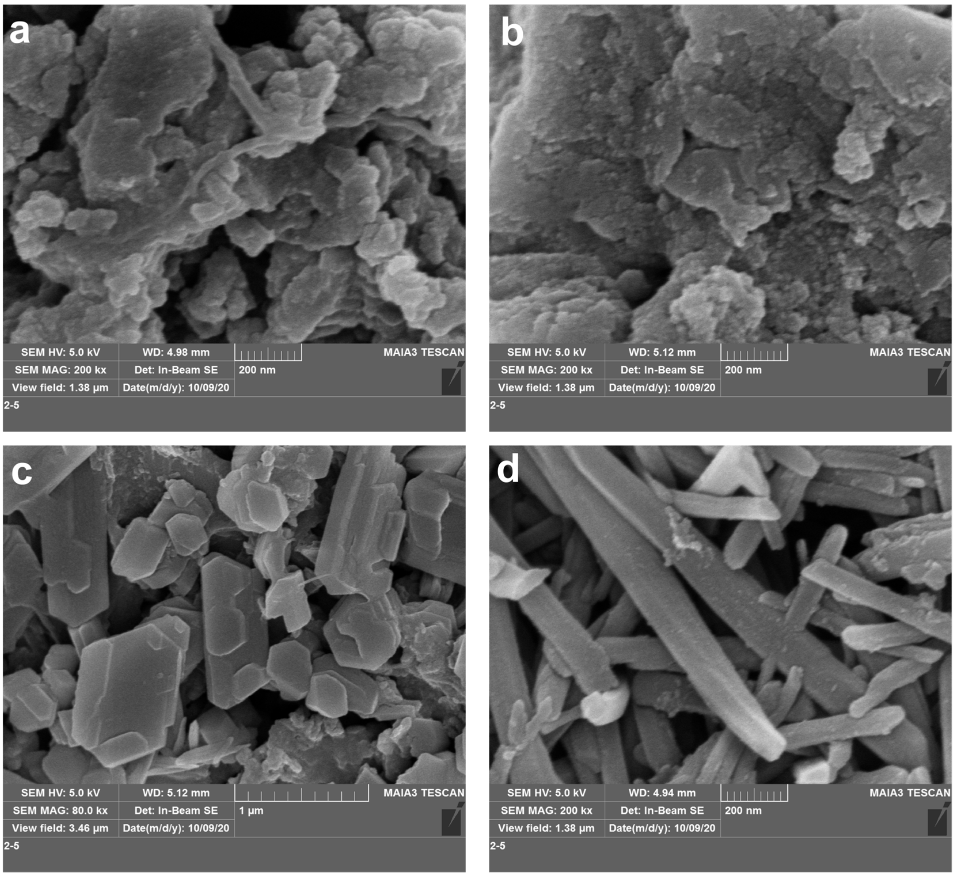

Supplementary Fig. 1. The SEM images of COP-Ene, COP-Ppcfe, COP-Nap and COP-Pyr samples.

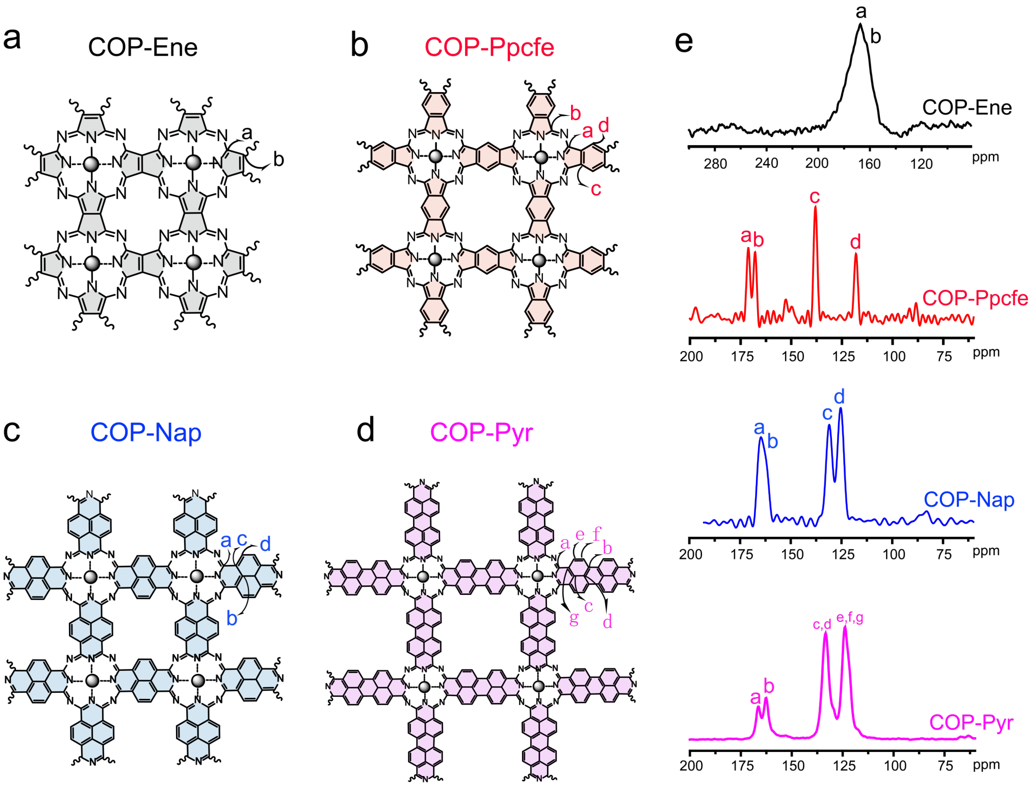

Supplementary Fig. 2. The ^{13}C solid-state nuclear magnetic resonance (NMR) spectra of COP-Ene, COP-Ppcfe, COP-Nap and COP-Pyr.

COP-Ene: ^{13}C NMR (MHz) δ 167.4 (a), δ 161.3 (b);

COP-Ppcfe: ^{13}C NMR (MHz) δ 171 (a), δ 167.4 (b), δ 138 (c), δ 118.3 (d);

COP-Nap: ^{13}C NMR (MHz) δ 165 (a), δ 162.3 (b), δ 131 (c), δ 125.5 (d);

COP-Pyr: ^{13}C NMR (MHz) δ 166 (a), δ 162.7 (b), δ 133.4 (c and d), δ 124 (e, f and g).

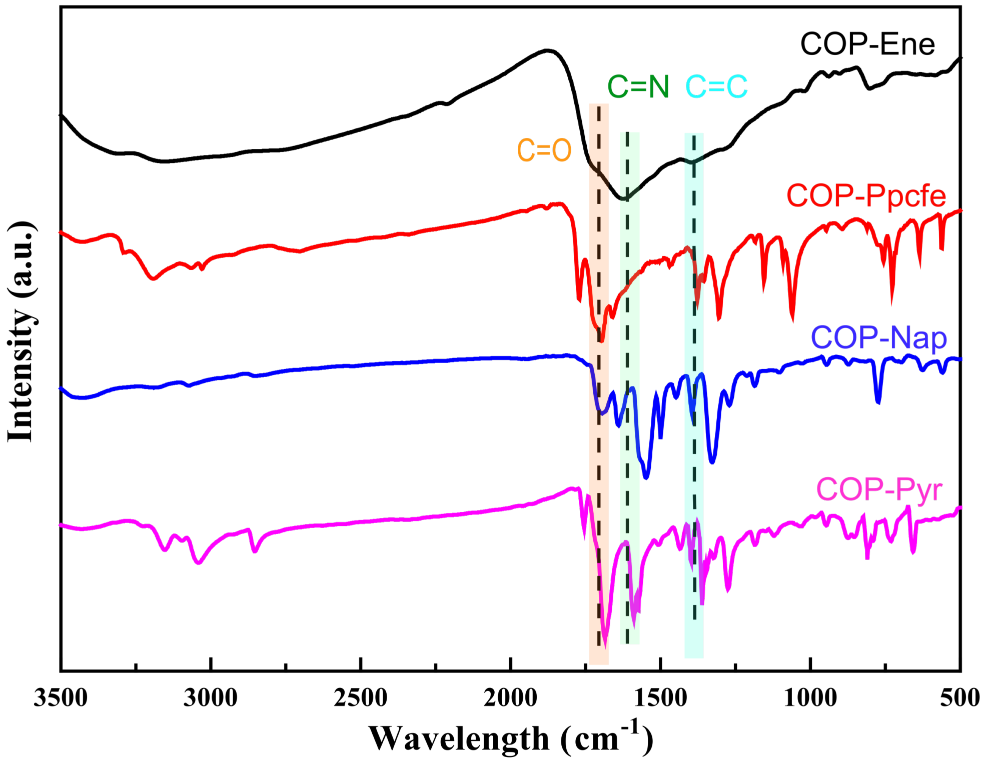

Supplementary Fig. 3. FT-IR spectra of COP-Ene, COP-Ppcfe, COP-Nap and COP-Pyr.

Notably, the FT-IR spectra of covalent organic polymers frameworks in all samples are presented the same signals: the stretching vibration peaks of ~1120 cm^{-1} region are assigned to the coordination bond between central metal and ring inner N in COPs; the stretching vibration peaks of ~900 cm^{-1} are ascrribed to metal ligand vibration; the stretching vibration peaks about 736 cm^{-1} region are ascrribed to the characteristic peaks of backbone. Peaks at ~1445 cm^{-1} region and ~1586 cm^{-1} region are ascribed to the stretching vibration of the C=C and C=N links connecting building block, respectively. The vibration peaks of C=O appear at ~1690 cm^{-1} and ~3046 cm^{-1}, respectively. Moreover, a sequential ascending C=C stretching vibrations (~1400 cm^{-1}) manifest successively incremental planar-electron abundance in for polymers. Through the peak position analysis of the above bonded groups comined with ^{13}C solid-state nuclear magnetic resonance (NMR) spectra (Supplementary Fig. 2), we proved that the four target samples were successfully synthesized.

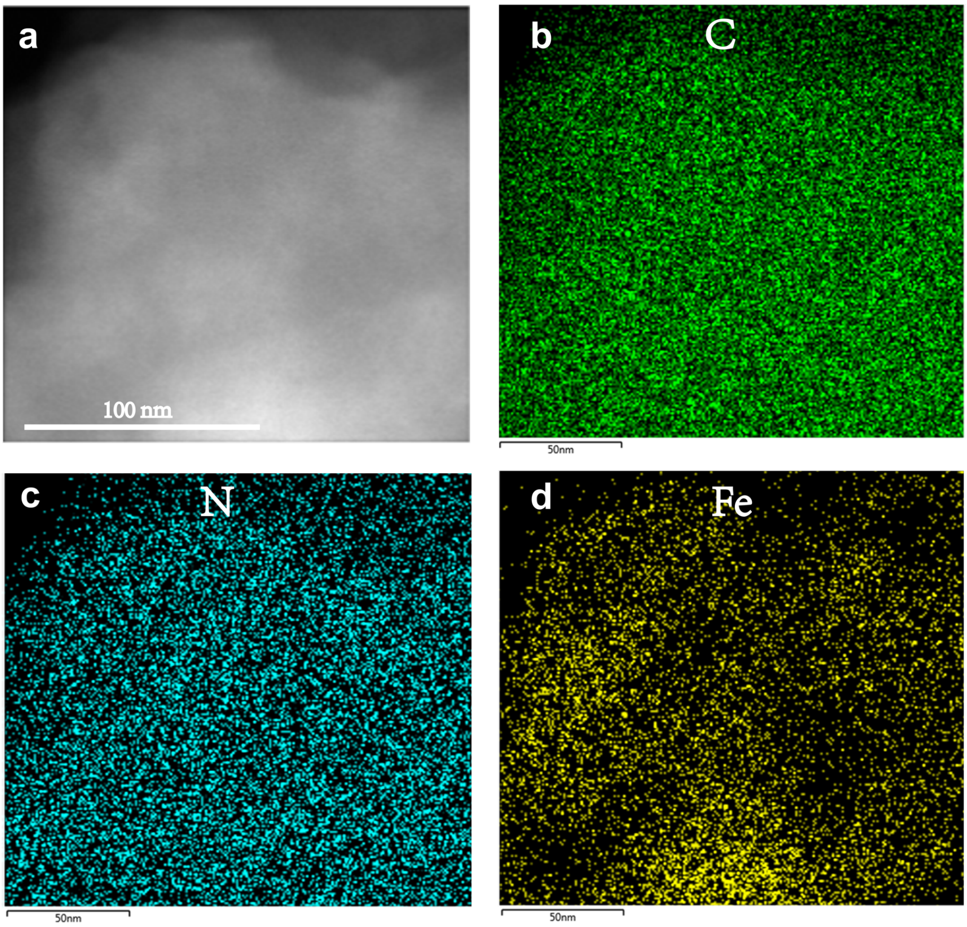

Supplementary Fig. 4. The EDX mapping images of COP-Ene sample in HRTEM.

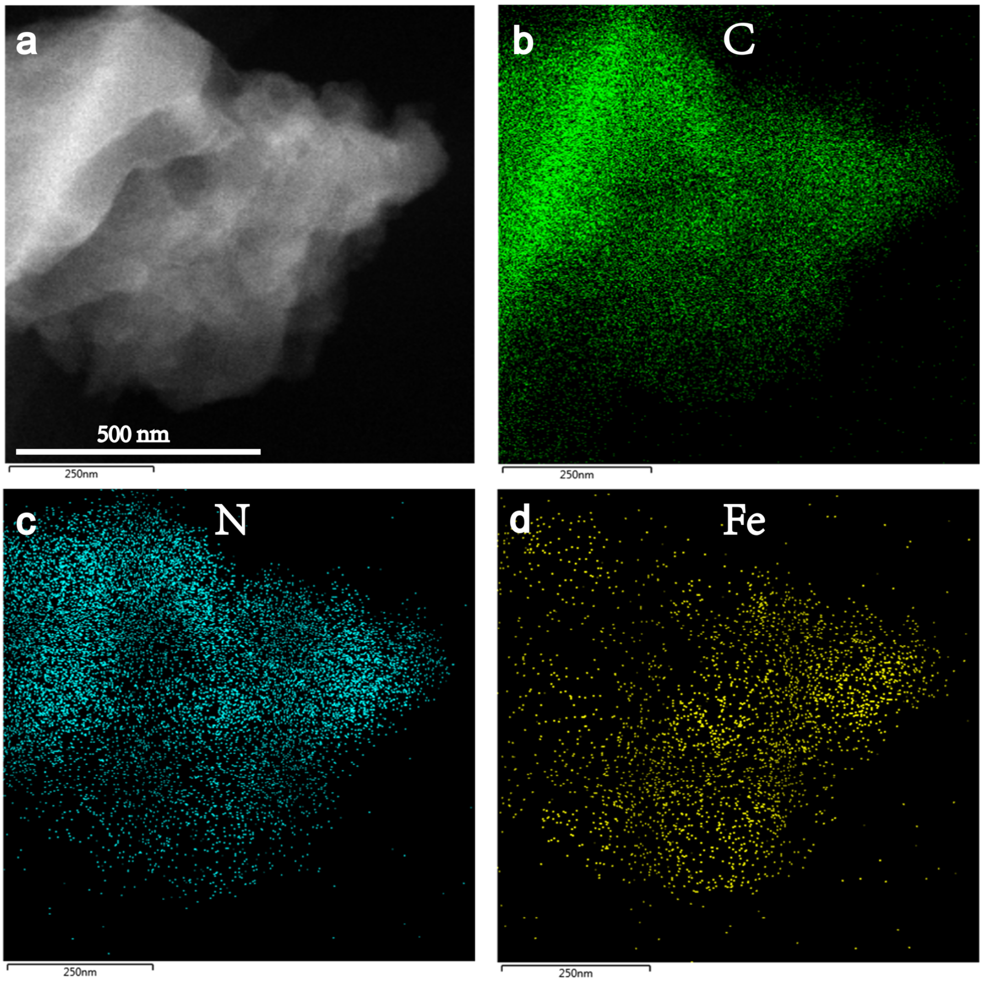

Supplementary Fig. 5. The EDX mapping images of COP-Ppcfe sample in HRTEM.

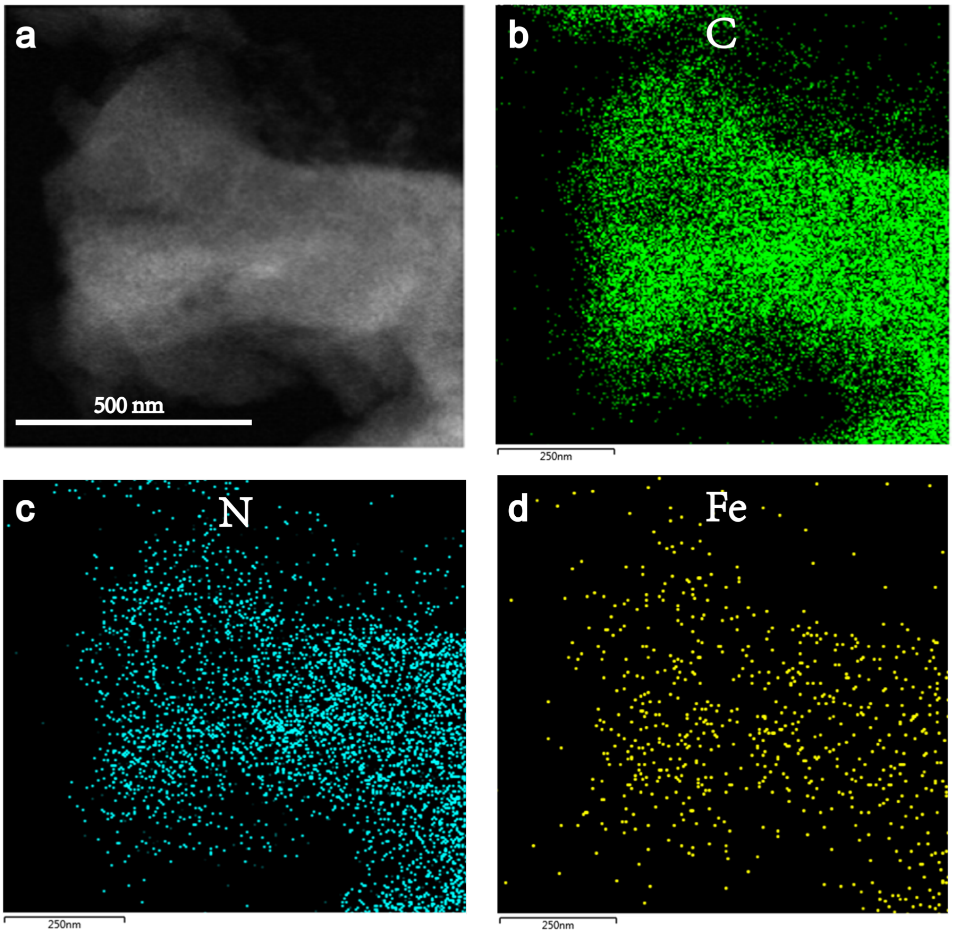

Supplementary Fig. 6. The EDX mapping images of COP-Nap sample in HRTEM.

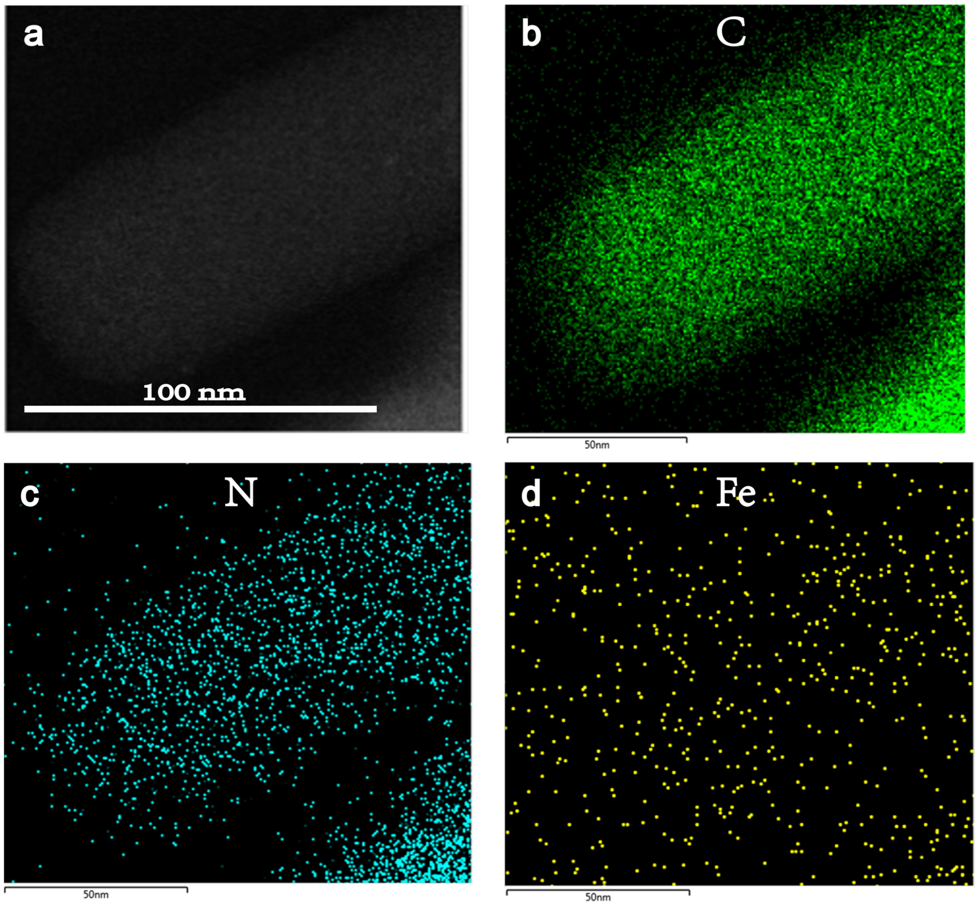

Supplementary Fig. 7. The EDX mapping images of COP-Pyr sample in HRTEM.

In the element mapping of Fe (Supplementary Figs. 4-7.), the good dispersibility of Fe atom not only effectively indicates that Fe exists as single iron atoms, but the gradual decrease in Fe atom content also reflects the gradual increase in the degree of conjugation of the carbon skeleton in the synthesized four sample.

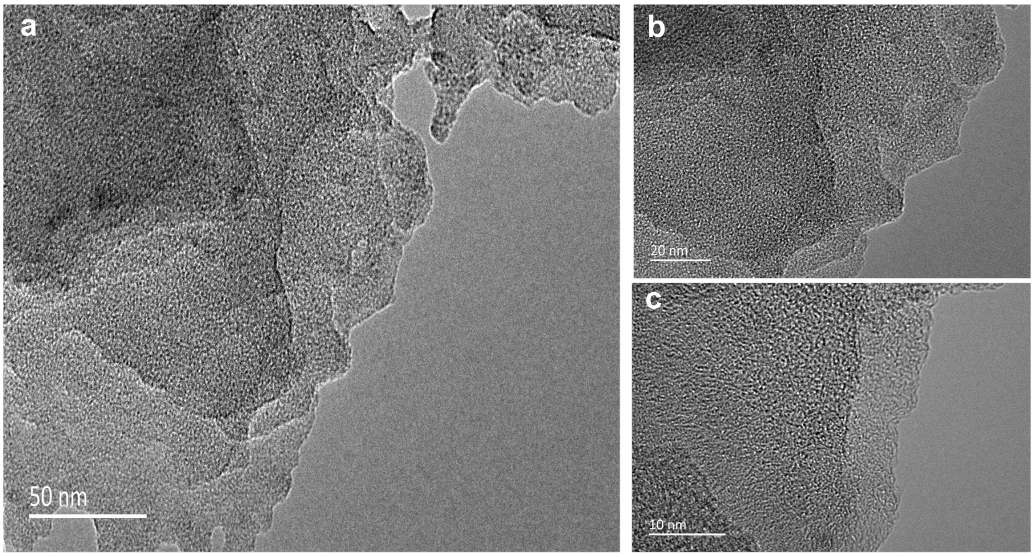

Supplementary Fig. 8. The HRTEM images of COP-Ppcfe sample.

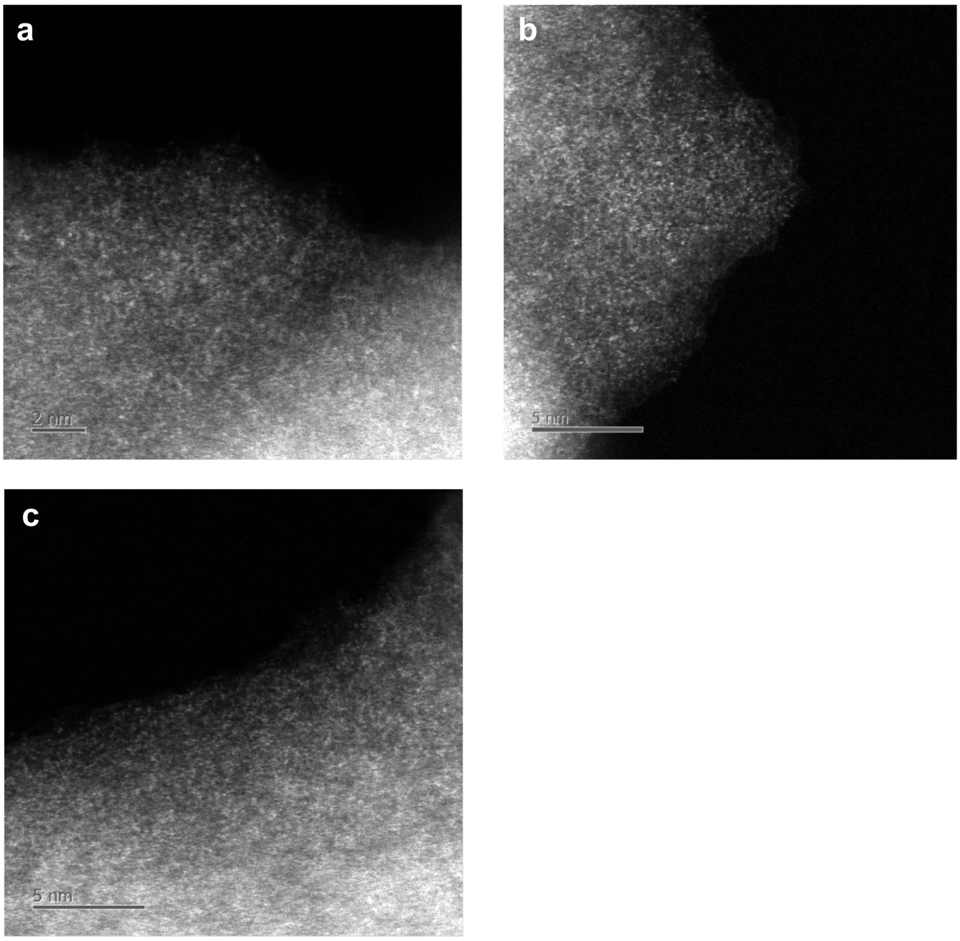

Supplementary Fig. 9. The HAADF STEM images of COP-Ppcfe sample at different positions.

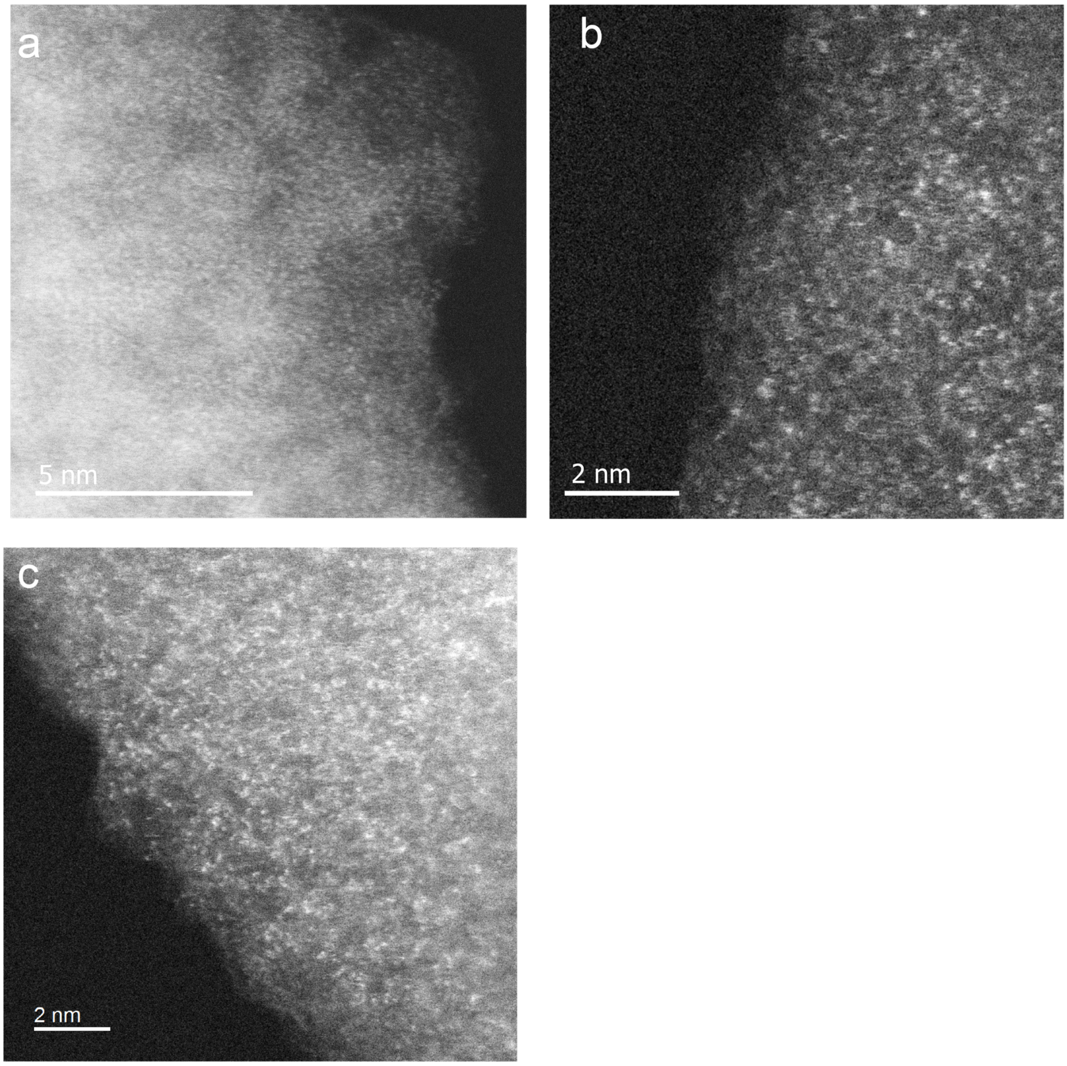

Supplementary Fig. 10. The HAADF STEM images of (a) COP-Ene, (b) COP-Nap and (c) COP-Pyr samples.

Through HRTEM, we observed that the Fe, N, and C in the COP-Ene, COP-Ppcfe, COP-Nap and COP-Pyr samples were uniformly dispersed, and no metal clusters were found. Furthermore, we observed multiple positions of the COP-Ene, COP-Ppcfe, COP-Nap and COP-Pyr samples through HAADF STEM, and observed numerous monodispersed bright spots images, which confirmed that the Fe atoms were dispersed in the form of atoms. In addition, the gradual decrease of Fe distribution contents also shows the gradual increase of the conjugated system in the four COPs, which is consistent with the Fe contents measured by ICP-OES.

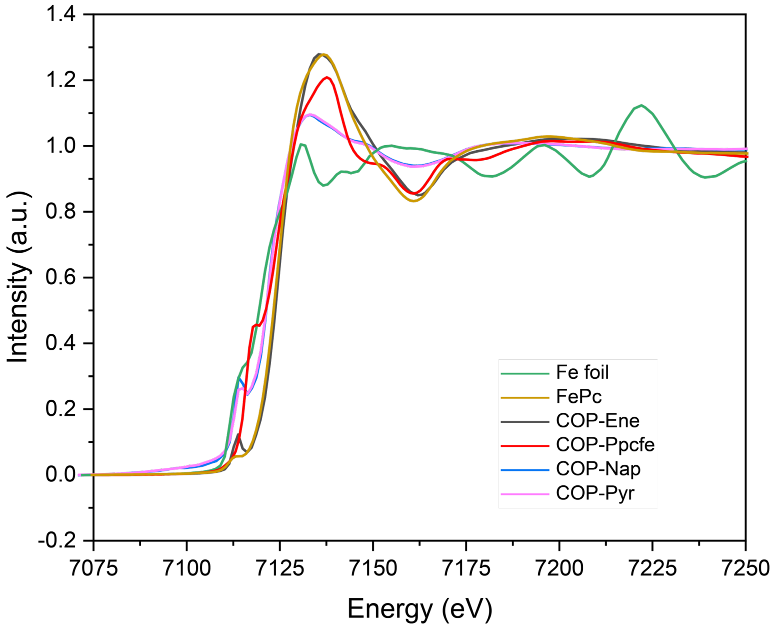

Supplementary Fig. 11. The Fe K-edge XANES of Fe foil, FePc and synthetic four samples.

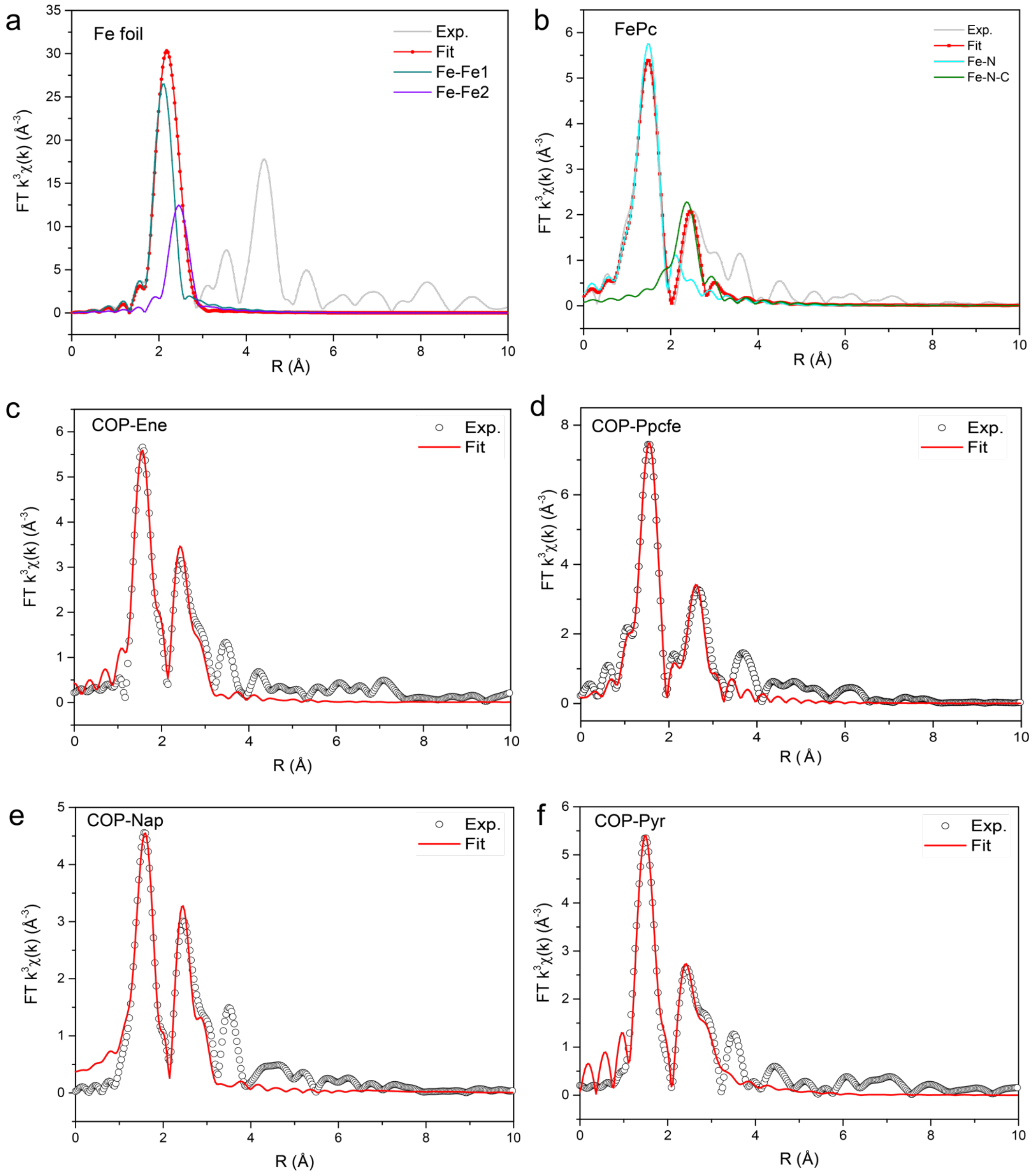

Supplementary Fig. 12. The Fourier transformations of the EXAFS spectra and corresponding EXAFS R space fitting curves of Fe foil, FePc and four synthetic samples.

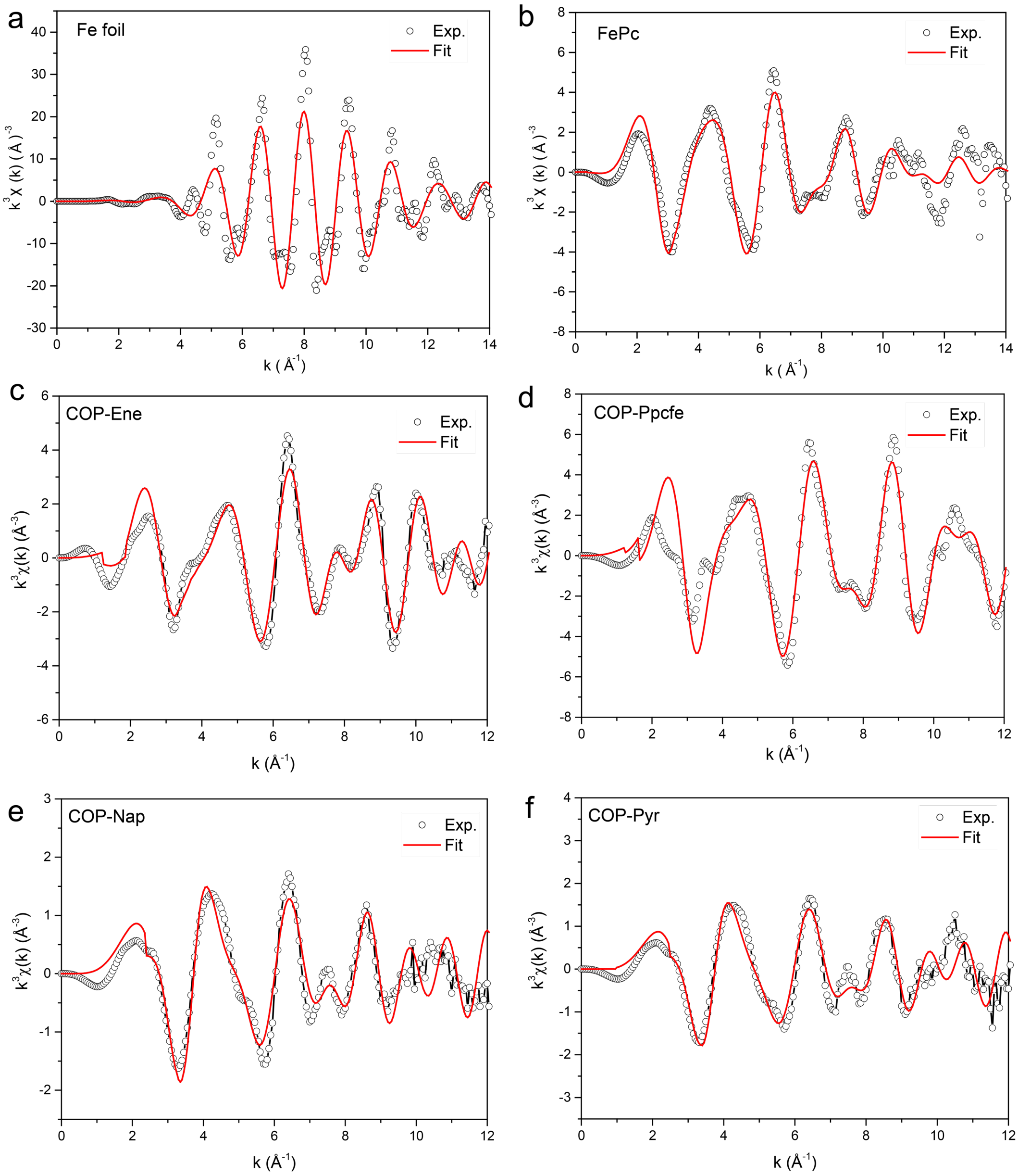

Supplementary Fig. 13. The corresponding EXAFS *k* space fitting curves of Fe foil, FePc and synthetic four samples.

Synthetic four samples were demonstrated high similarity to FePc benchmark, exhibiting dominated peaks corresponding to the Fe-N (~ 1.53 Å) and Fe-N-C (~ 2.7 Å) scattering paths, whereas the Fe-Fe bond (~ 2.18 Å) was not observed (Fig. 2d and Supplementary Fig. 12). EXAFS fitting of four sample in k-space were also consistent with that of FePc benchmark, whereas completely deviated from Fe foil (Fig. 2e, Supplementary Fig. 13), manifesting iron atom existing as mononuclear centers. The Fe K-edge XANES profiles in Supplementary Fig. 11 suggest the oxidation valence state of the isolated single Fe atoms in four COPs is likely to be higher than metallic

Fe^0 (Fe foil) and lower than Fe^{2+} (FePc), which indicates that the Fe-N_4 structure in four COP samples adsorbed O_2 of the air during storage or testing.[1,2]

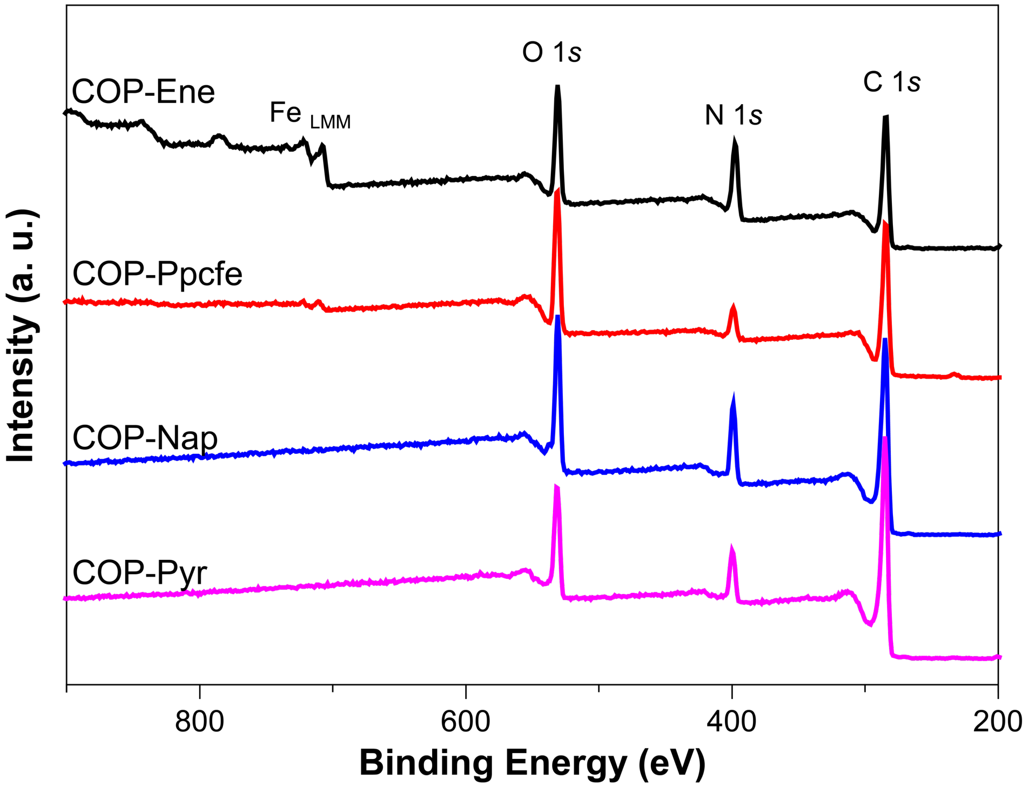

Supplementary Fig. 14. XPS survey spectra of four samples with COP-Ene, COP-Ppcfe, COP-Nap and COP-Pyr.

The gradually weakening of Fe_{LMM} peak with the augment of the carbon conjugated skeletons also suggests stronger conjugated degree of the polymerization skeletons.

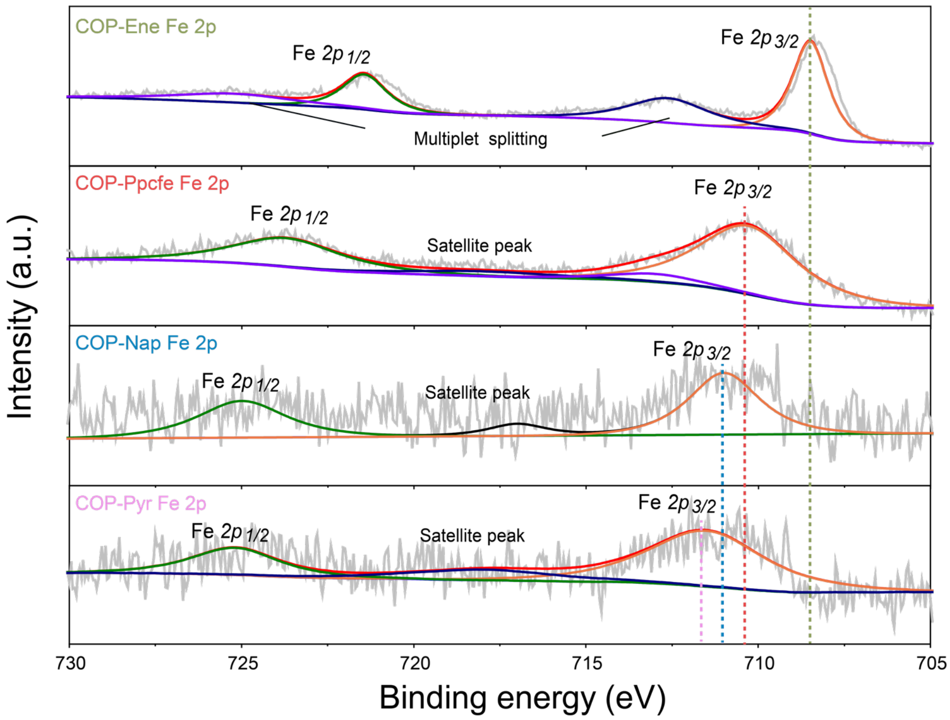

Supplementary Fig. 15. The Fe 2p XPS spectra of COP-Ene, COP-Ppcfe, COP-Nap and COP-Pyr.

The increasing DDE in the carbon matrix adjacent to FeN_4 moieties makes Fe 2$p_{1/2}$ and Fe 2$p_{3/2}$ peaks in COP-Nap and COP-Pyr samples closer to the higher binding energy than COP-Ene and COP-Ppcfe.

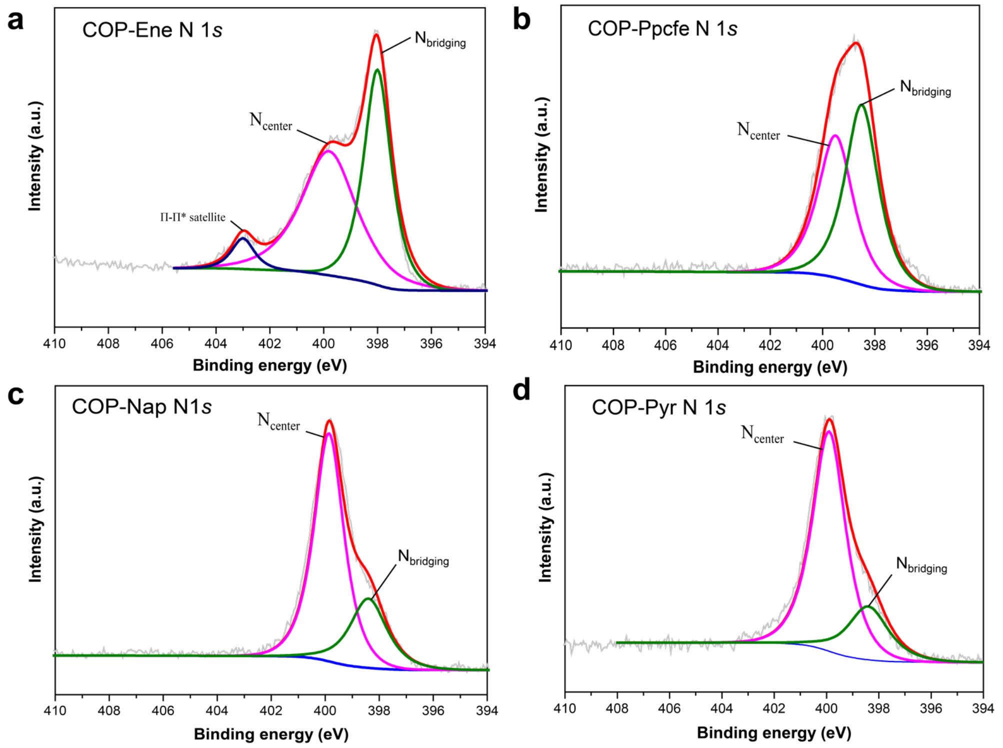

Supplementary Fig. 16. The N1*s* XPS spectra of (a) COP-Ene; (b) COP-Ppcfe; (c) COP-Nap; (d) COP-Pyr.

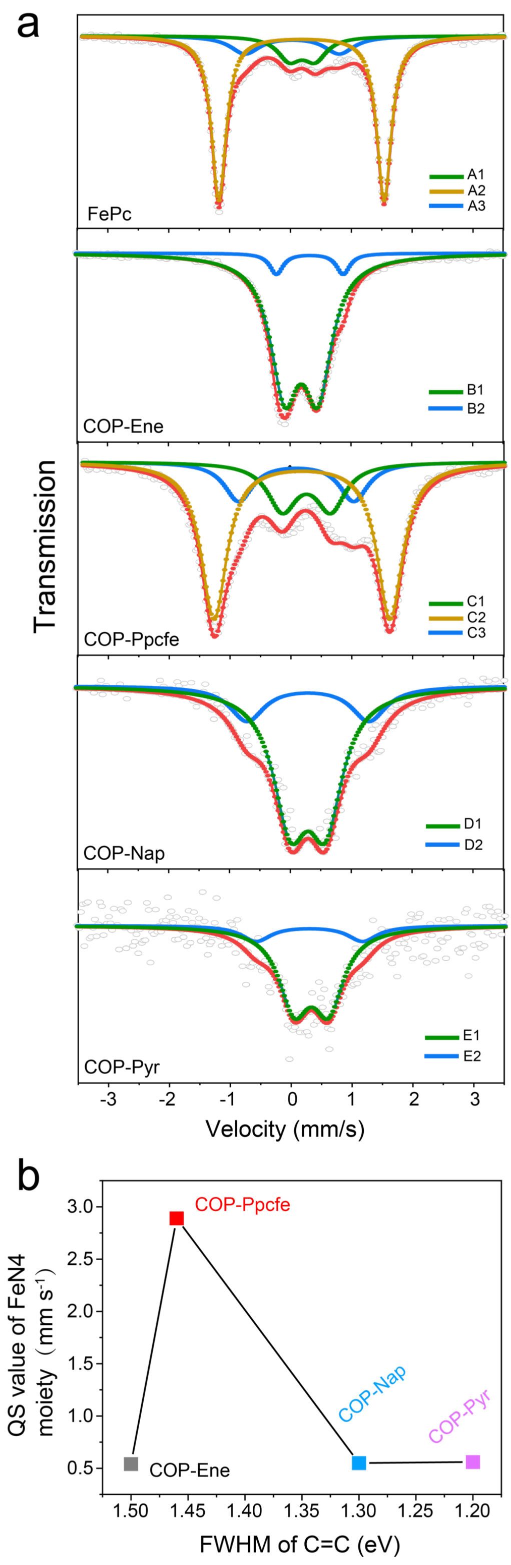

Supplementary Fig. 17. (a) Room-temperature ^{57}Fe Mössbauer spectra of FePc, COP-Ene, COP-Ppcfe, COP-Nap and COP-Pyr samples, respectively. (b) Volcano plot between the structural change of FeN_4 site in four samples (quantified by QS value of doublet FeN_4 moiety) and DDE (quantified by FWHM).

We tested four samples and the benchmark FePc (directly bought from *Macklin Biochemical Co., Ltd.*) and fitted Mössbauer curves. We keep two significant digits after the decimal point for all parameters of the fitting for Mössbauer spectrums in Supplementary Table 3. The FePc is fitted to three doublets. The relevant literatures show that the doublet A2 can be attributed to the FeN_4 site and the doublet A1 and A3 can be attributed to iron microenvironments absorbed oxygen between the layers of iron phthalocyanines (*J. Phys. Chem. 1980, 84, 1936-1939; Hyperfine Interactions 139/140: 631-639, 2002.*). The difference between the doublet A1 and A3 may be due to the molecular stacking arrangements between FePc molecules, resulting in small differences in the interaction with oxygen. FeN_4 moieties is generally recognized to be greatly easy to adsorb oxygen, therefore, two or three doublets detected by the Mössbauer spectroscopy for a sample with the single pure component may be inevitable due to the extremely high sensitivity to energy changes on the order of 10^{-8} eV (ca. 10^{-4} cm^{-1}) and extreme sharpness of tuning (ca. 10^{-13}) for Mössbauer spectroscopy. Because building blocks in our COP-Ppcfe sample extremely resemble that of the benchmark FePc, the test results about COP-Ppcfe sample are also greatly consistent with that of the FePc. Therefore, the doublets may be assigned as: the doublet C2 to FeN_4 site, the doublet C1 and doublet C3 to a small amount of iron microenvironments containing oxygen between the layers of iron phthalocyanines. The difference between the doublet C1 and C3 may be due to the staggered layers in COP-Ppcfe resulting in a slight difference with adsorbed oxygen. As for COP-Nap and COP-Pyr samples, from the molecular structure (pyridine N connected to Fe instead of pyrrole N) and ORR test results (similar LSV curves, *vice infra*), we can predict that their active sites should be particularly similar in theory. In our test results, the isomer shift (IS) and quadrupole splitting (QS) values are similar, which is consistent with theory. Combined with result of XAFS, we attribute main doublet D1 and doublet E1 to FeN_4 sites. For doublet D2 and doublet E2, because of their QS values similar to doublet A3 sites in FePc, they also may be attributed to iron microenvironments containing absorbed oxygen between the layers of COP. Similarly, for COP-Ene, doublet B1 may be attributed to FeN_4 site, and doublet B2 may be attributed to FeN_4 moiety absorbed oxygen. In our samples, the

changes about IS (the maximum value of 0.33 mm s^{-1} in E1, the minimum value of 0.03 mm s^{-1} in A3) are nondiscriminating. For the deviation about QS between the doublet B2 site (QS=1.09) in COP-Ene and the doublet D2 site (QS=1.74) in COP-Nap, this change may be caused by the changes in the degree of delocalization of the benzene ring adjacent to FeN_4 sites, which is similar to the case of FeN_4C_8 and FeN_4C_{10} moieties in pyrolyzed Fe-N-C catalyst (*Energy Environ. Sci. 2019, 12, 2548-2558*). In addition, doublet FeN_4 moiety in COP-Ene, COP-Nap and COP-Pyr exhibit different QS values compared to COP-Ppcfe (0.54 mm s^{-1} for COP-Ene, 0.55 mm s^{-1} for COP-Nap, 0.56 mm s^{-1} for COP-Pyr *vs.* 2.89 mm s^{-1} for COP-Ppcfe), reflecting that DDE in the carbon matrix induces the changes of electronic configuration of FeN_4 moiety in four samples.

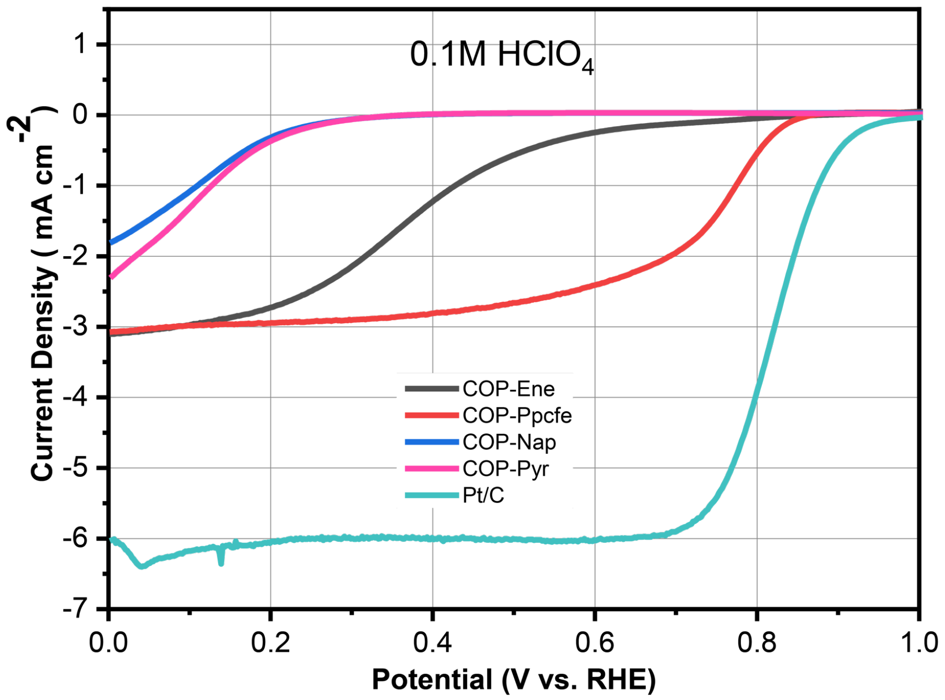

Supplementary Fig. 18. LSV curves of COP-Ene, COP-Ppcfe, COP-Nap, COP-Pyr samples and Pt/C in O_2-saturated 0.1 $HClO_4$ solution at a scan of 5 mV s^{-1} and a rotation speed of 1600 rpm.

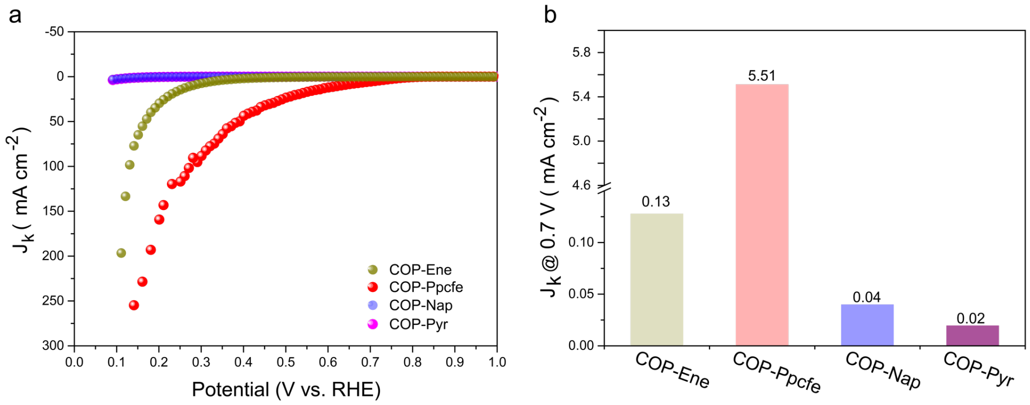

Supplementary Fig. 19. (a) Kinetic current density (J_k) of COP-Ene, COP-Ppcfe, COP-Nap and COP-Pyr over 0 ~ 1 V versus RHE. (b) The kinetic current density (J_k) @ 0.7 V of COP-Ene, COP-Ppcfe, COP-Nap and COP-Pyr.

The kinetic current density (J_k) @ 0.7 V embodies discrepancy of as-synthesis catalysts in electrocatalytic kinetics. As observed by Supplementary Fig. 19, the COP-Ppcfe sample exhibits a superior J_k of 5.51 mA cm^{-2} at 0.7 V (versus RHE), which was about 42 times higher than that of COP-Ene, 137 times higher than that of COP-Nap and 275 times higher than that of COP-Pyr in 0.1 M $HClO_4$. These results indicate that the kinetics of the four COPs also show a trend of increasing first and then decreasing with the increase of the DDE.

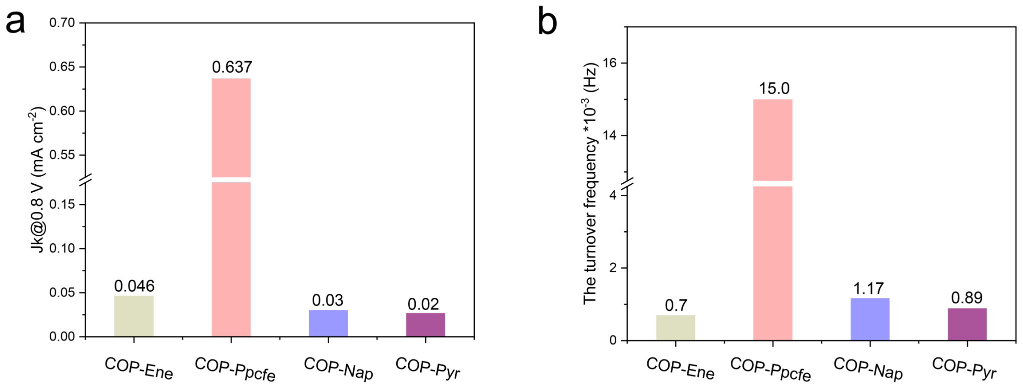

Supplementary Fig. 20. (a) The kinetic current density (J_k) @ 0.8 V of COP-Ene, COP-Ppcfe, COP-Nap and COP-Pyr. (b) The turnover frequency at 0.8 V of COP-Ene, COP-Ppcfe, COP-Nap and COP-Pyr.

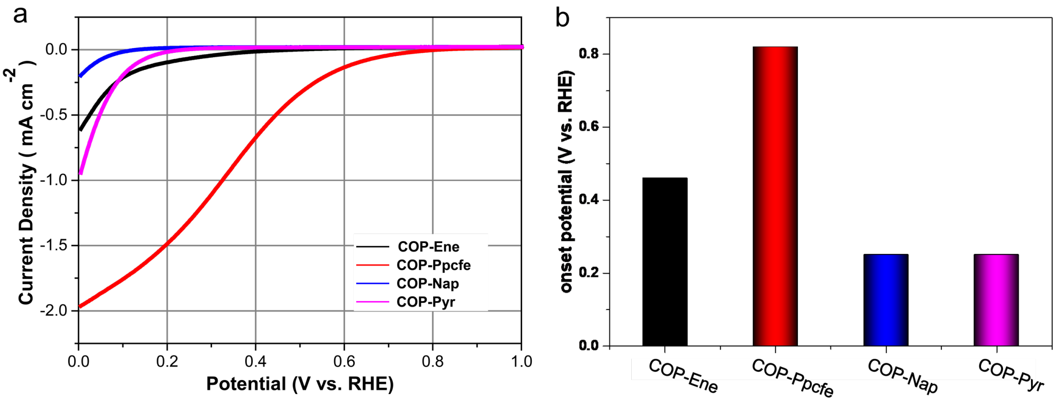

Supplementary Fig. 21. Electrochemical characterization. (a) LSV curves (b) onset potential of COP-Ene, COP-Ppcfe, COP-Nap and COP-Pyr without Cabot Vulcan XC-72 in O_2-saturated 0.1 $HClO_4$ solution at a scan of 5 mV s^{-1} and a rotation speed of 1600 rpm.

To exclude the influence of the ORR activity of the conductive agent XC72, we tested their ORR activity without the conductive agent in 0.1M $HClO_4$. The activity trend (initial potential) of the different samples remained consistent with the condition of carbon black.

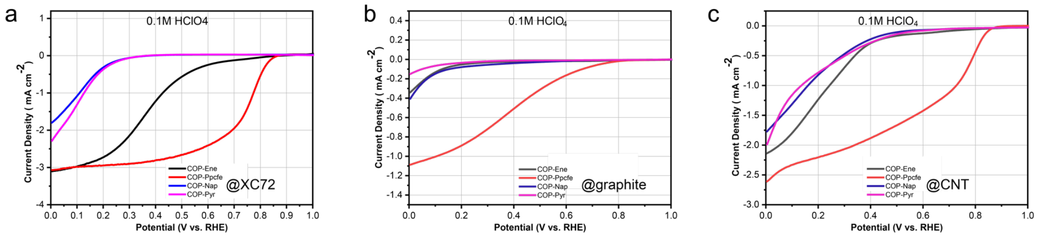

Supplementary Fig. 22. a LSV curves of COP-Ene, COP-Ppcfe, COP-Nap, and COP-Pyr samples loaded with XC-72. b LSV curves of COP-Ene, COP-Ppcfe, COP-Nap, and COP-Pyr samples loaded with graphite in O_2-saturated 0.1 $HClO_4$ solution. c LSV curves of COP-Ene, COP-Ppcfe, COP-Nap, and COP-Pyr samples loaded with CNT in O_2-saturated 0.1 $HClO_4$ solution.

We considered that the introduction of carbon supports (XC-72) may produce DDE effects, so we did an acidic ORR experiment with pure COP materials without foreign

carbon supports, and made corresponding discussions (Supplementary Fig. 22). It can be observed that although the ORR performance of the four COPs materials in acidic medium is very low, their onset potential still satisfies the volcanic curve relationship. In fact, considering the limited conductivity of these materials, we only added the carbon support XC-72 in the electrochemical test instead of in the experimental synthesis process and the carbon support is added with the same mass under the same conditions. The purpose of this experimental operation is to increase the conductivity of the catalyst while minimizing the interaction between the carbon support and the COPs, that is, to eliminate the DDE effect of carbon supports. In order to further eliminate the effect of the addition of carbon supports on COP's materials, besides hard carbon (XC-72, typical hard carbon) loaded COP materials, we additionally use soft carbon (graphite) and rigid carbon (carbon nano tube, CNT) as supports, and found the same law: regardless of the type of carbon support supported by these polymers, their catalytic activity of these polymers exhibits a volcano relationship.

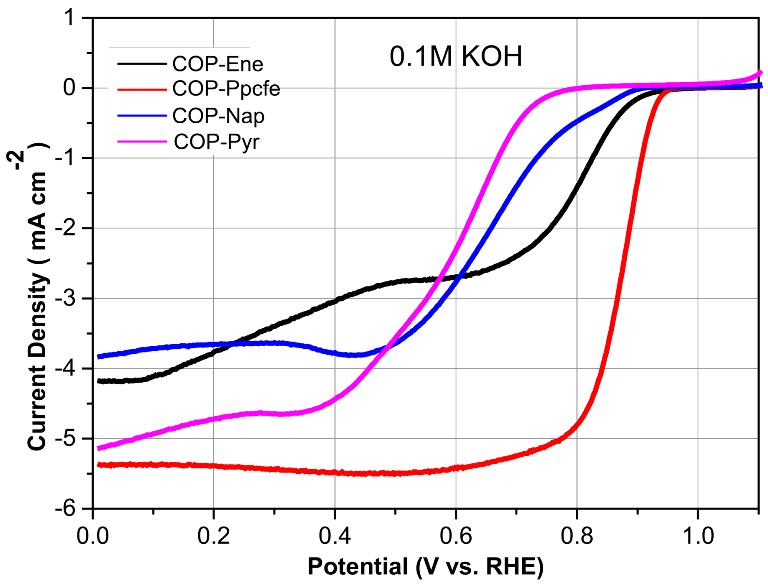

Supplementary Fig. 23. Electrochemical characterization. LSV curves of COP-Ene, COP-Ppcfe, COP-Nap and COP-Pyr samples in O_2-saturated 0.1 KOH solution at a scan of 5 mV s^{-1} and a rotation speed of 1600 rpm.

To distinguished COP-Nap and COP-Pyr sample oxygen reduction activity, the ORR measurements for four COPs were first carried out in 0.1 M KOH (mass loading of about 0.255 mg cm^{-2}). Linear sweep voltammetry (LSV) curves in Supplementary Fig. 23 exhibit their halfwave potential ($E_{1/2}$) comparison, following the order of COP-Ppcfe ($E_{1/2}$=874 mV) > COP-Ene ($E_{1/2}$=799 mV) > COP-Nap ($E_{1/2}$=669 mV) > COP-Pyr ($E_{1/2}$=598 mV), and embody a volcano relationship.

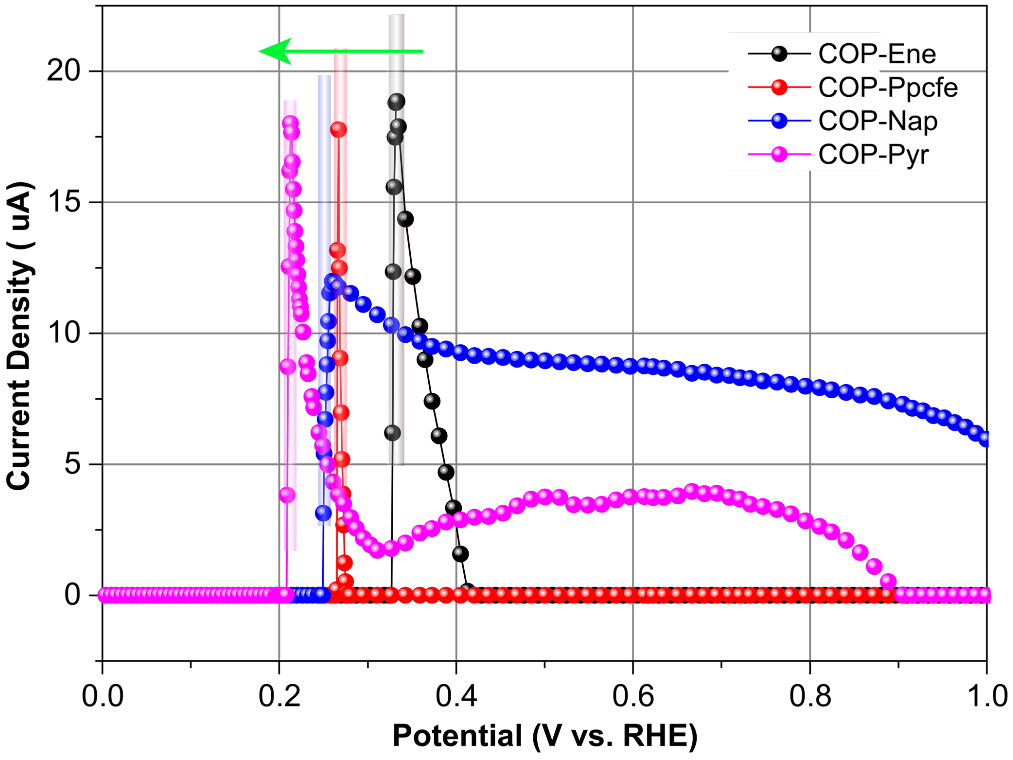

Supplementary Fig. 24. Square wave voltammetry (SWV) profiles of COP-Ene, COP-Ppcfe, COP-Nap and COP-Pyr samples.

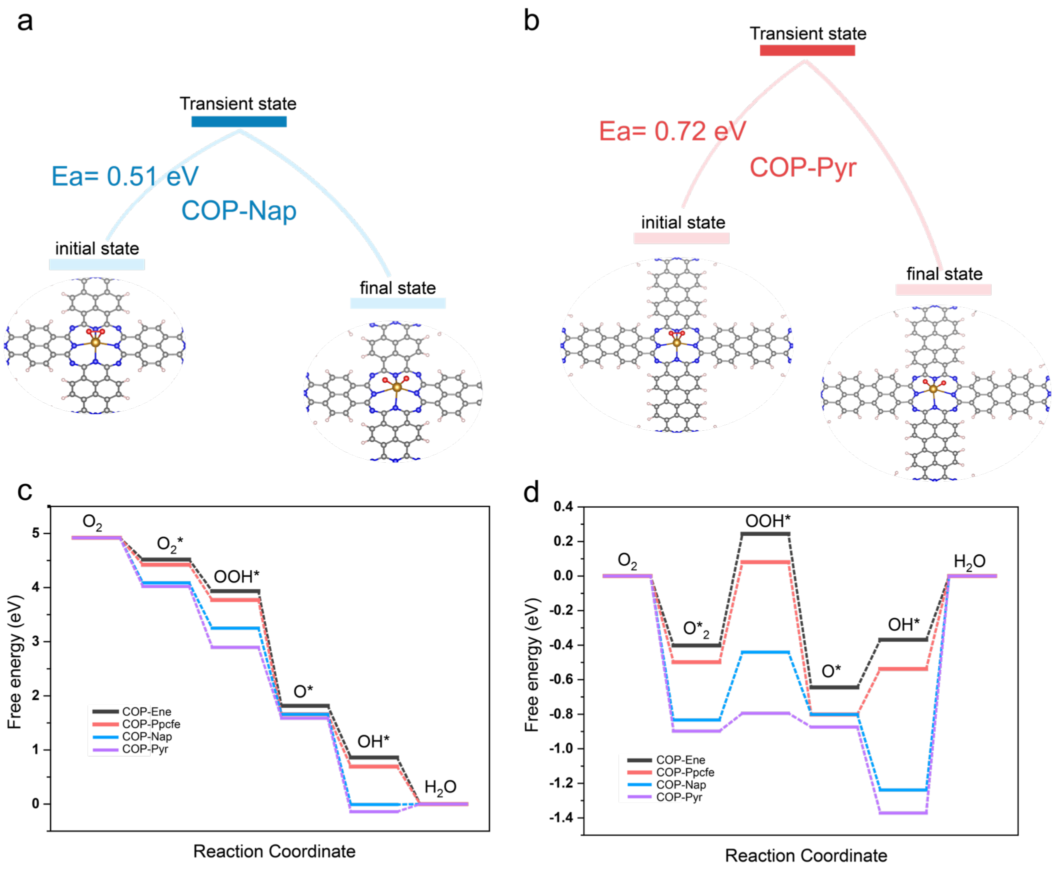

Supplementary Fig. 25. (a) Calculated activation energies for COP-Nap in dissociative mechanism. (b) Calculated activation energies for COP-Pyr in dissociative mechanism. (c) Free energy diagram for ORR on COP-Ene, COP-Ppcfe, COP-Nap and COP-Pyr at zero electrode potential. (d) Free energy diagram for ORR on COP-Ene, COP-Ppcfe, COP-Nap and COP-Pyr at equilibrium electrode potential.

Because COP-Nap and COP-Pyr taken a side-on configuration for dioxygen adsorption, we calculated the activation energies of O_2^* dissociation, corresponding to 0.51 eV and 0.72 eV for COP-Nap and COP-Pyr, respectively. The grand barriers means that O_2 dissociative mechanism for COP-Nap and COP-Pyr is not considered.

In the associative mechanism, O_2 activation ($O_2^* + H^+ + e^- \rightarrow OOH^*$) in COP-Ene is the rate-determining step of four-electron reaction. When an electrode potential of U= 0.5836 V is applied, all the elementary steps of the oxygen reduction reaction in COP-Ene can occur spontaneously. Interestingly, COP-Ppcfe can co-balance the adsorption

for the multiple reaction intermediates in oxygen reduction process, where the Gibbs free energies required during O_2^* activation and OH* desorption are roughly equal. On the contrary, the sudden oxytropism of the central metal in the COP-Nap and COP-Pyr impose an obstacle to occur desorption of OH* intermediate, which limits the subsequent occurrence of entire four-electron reaction.

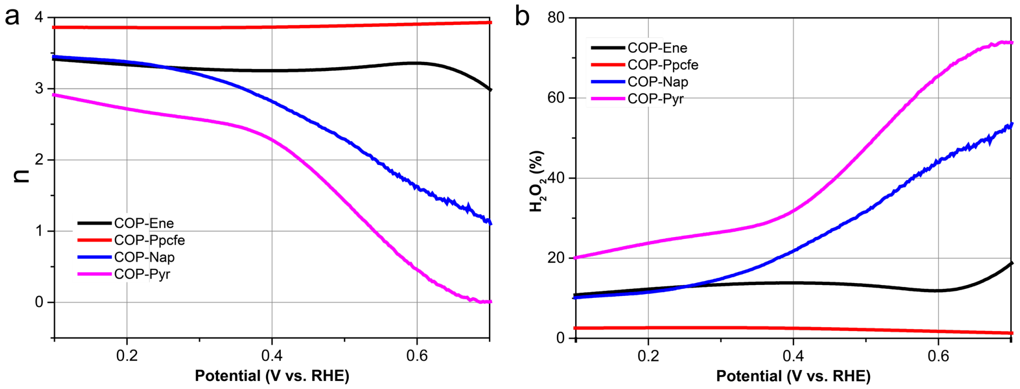

Supplementary Fig. 26. The electrode transfer numbers and H_2O_2 yields of COP-Ene, COP-Ppcfe, COP-Nap and COP-Pyr in O_2-saturated 0.1 $HClO_4$ solution at a scan of 5 mV s^{-1} and a rotation speed of 1600 rpm.

The COP-Ppcfe sample is close to the four-electron reaction mechanism during the electrochemical reaction, and the H_2O_2 yield is less than 5%. For COP-Ene sample, the number of electrons transferred is close to between four electrons and two electrons, and about 10% of H_2O_2 is generated. However, the COP-Nap and COP-Pyr samples exhibited almost complete two-electron reaction under high voltage, and the H_2O_2 yield exceeded 50%.

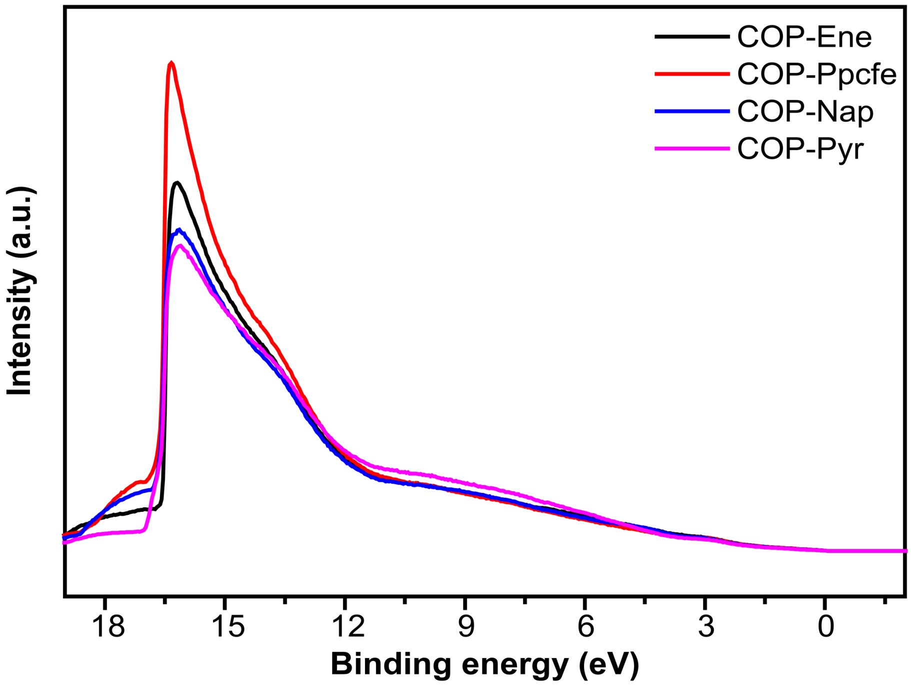

Supplementary Fig. 27. The UPS of four samples (COP-Ene, COP-Ppcfe, COP-Nap and COP-Pyr).

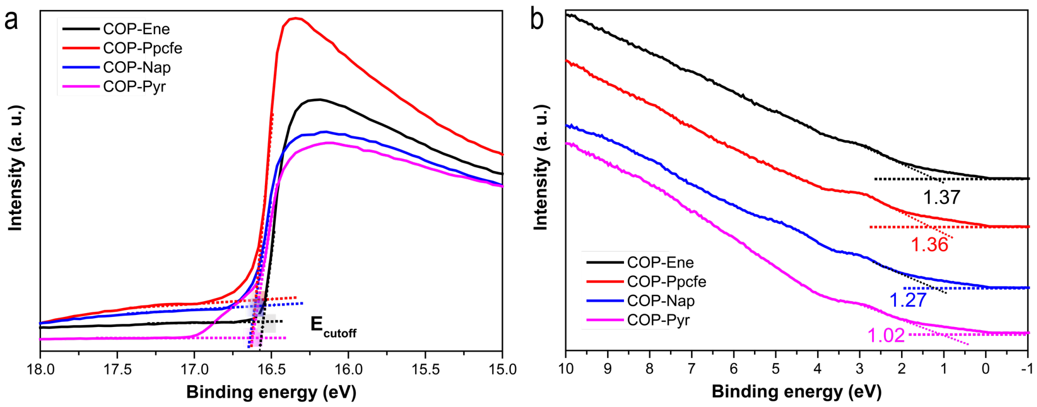

Supplementary Fig. 28. (a) UPS work function (φ) spectra of COP-Ene, COP-Ppcfe, COP-Nap and COP-Pyr. (b) the UPS valence band spectra of COP-Ene, COP-Ppcfe, COP-Nap and COP-Pyr.

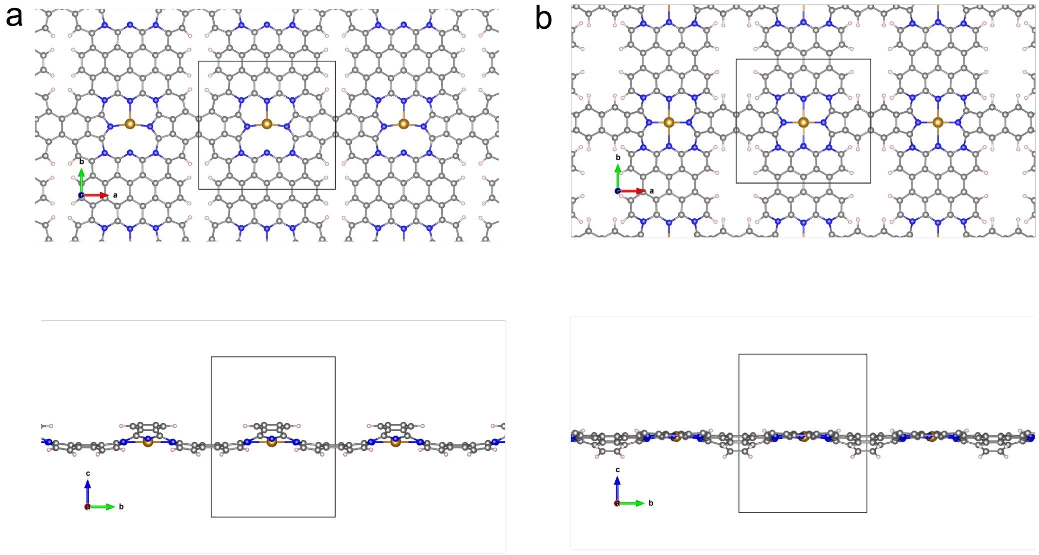

Supplementary Fig. 29. The structure obtained by continuously incremental the π-conjugated system based on COP-Pyr configuration.

From COP-Ene to COP-Pyr model, the degree of delocalization stemmed from π-electrons of the carbon conjugated skeleton around the FeN_4 site is progressively incremental. When we try to continue to increase the π conjugated moieties, the model of macrocyclic structure exhibits a certain degree of skeleton bending, indicating that further incremental conjugated structures will reduce the stability of polymers.

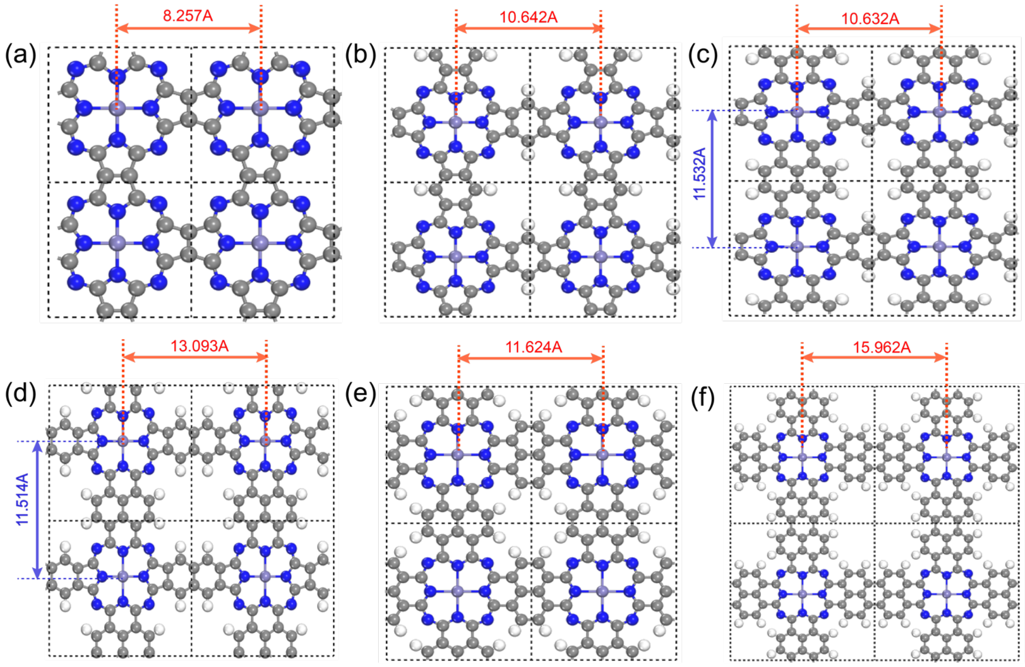

Supplementary Fig. 30. Schematic diagram of the Fe-Fe bond length in the six proposed models. The purple, dark blue, white, and grey colors represent Fe, N, H, and C atom, respectively.

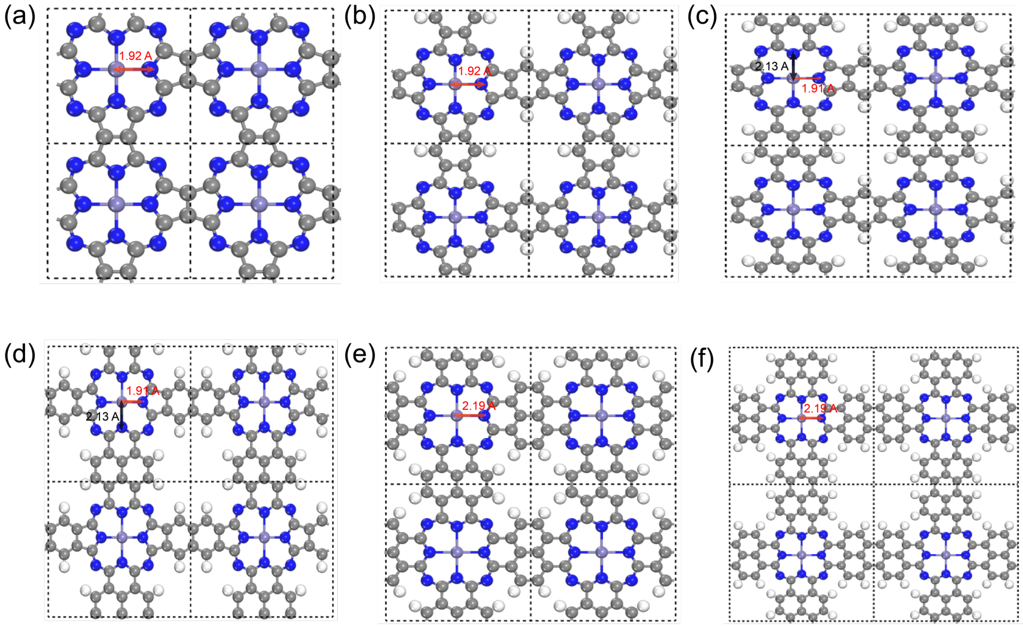

Supplementary Fig. 31. Schematic diagram of the Fe-N bond length in the six proposed models. The purple, dark blue, white, and grey colors represent Fe, N, H, and C atom, respectively.

In the proposed six models, there is no direct rule for the bond length of Fe-Fe bond or Fe-N bond with the expansion of benzene ring. Therefore, some influence factors towards ORR performance such as single atom density or nitrogen atom types can be overlooked.

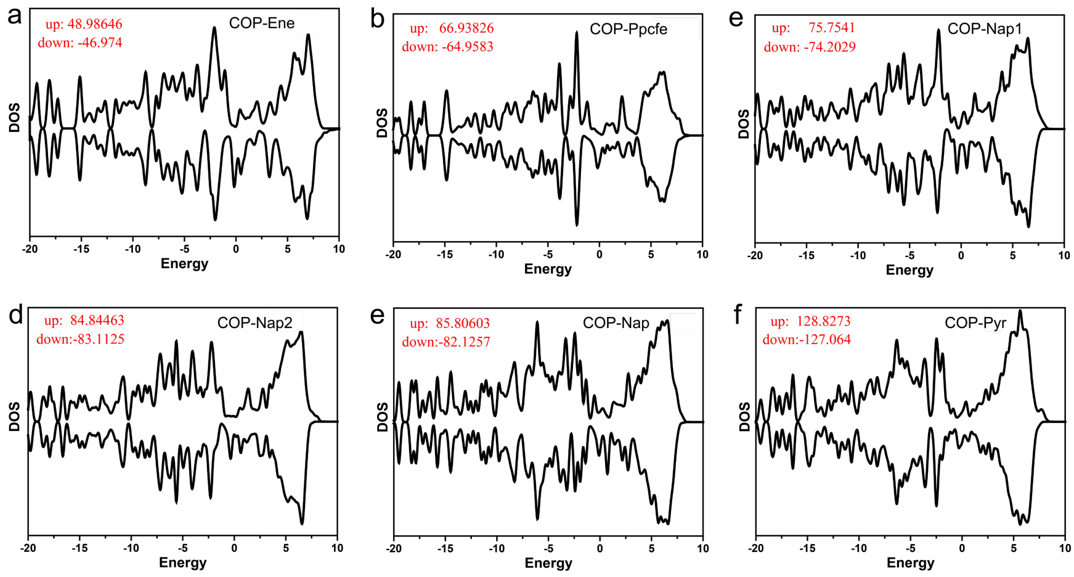

Supplementary Fig. 32. The total density of states (TDOS) of COP-Ene, COP-Ppcfe, COP-Nap1, COP-Nap2, COP-Nap and COP-Pyr models.

In the macrocyclic structure species, as the conjugated skeleton increases, the number of electrons delocalized in the carbocyclic skeleton in the system gradually increases, regardless of whether the electrons spin up or spin down, which corresponds with the conclusion obtained experimentally by FWHM (Fig. 2g-h).

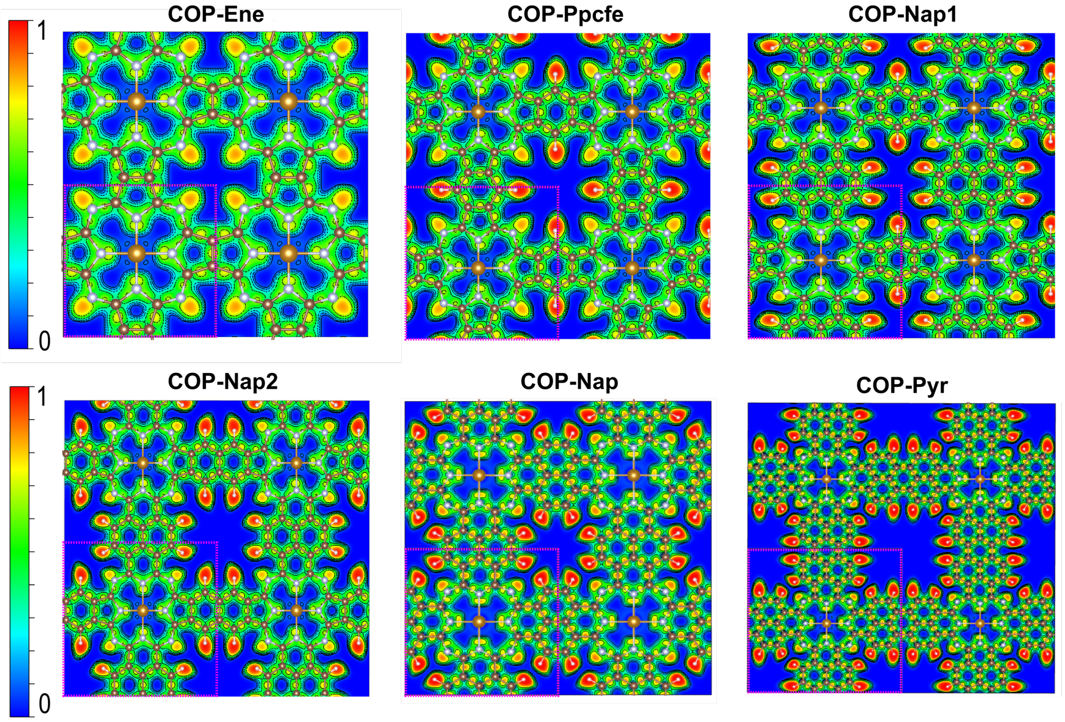

Supplementary Fig. 33. The Electron localization functions (ELF) diagrams of different models (COP-Ene, COP-Ppcfe, COP-Nap1, COP-Nap2, COP-Nap, COP-Pyr). The primitive cell is inside red dotted curve and the entire graph is a 2*2 supercell.

It can be seen from the ELF diagram that electrons are mainly delocalized in the C=C or C=N carbon skeleton. And with the expansion of the carbon skeleton adjacent to the FeN_4 site in the four COPs, the area of electron delocalization in the carbon matrix also gradually increases, which corresponds with the conclusion obtained experimentally by FWHM (Fig. 2g-h). The ELF values of the carbon skeletons (C=C or C=N) in all synthesized COPs are between 0 and 1, reflecting the existence of carbon atoms in the form of covalent bonds.

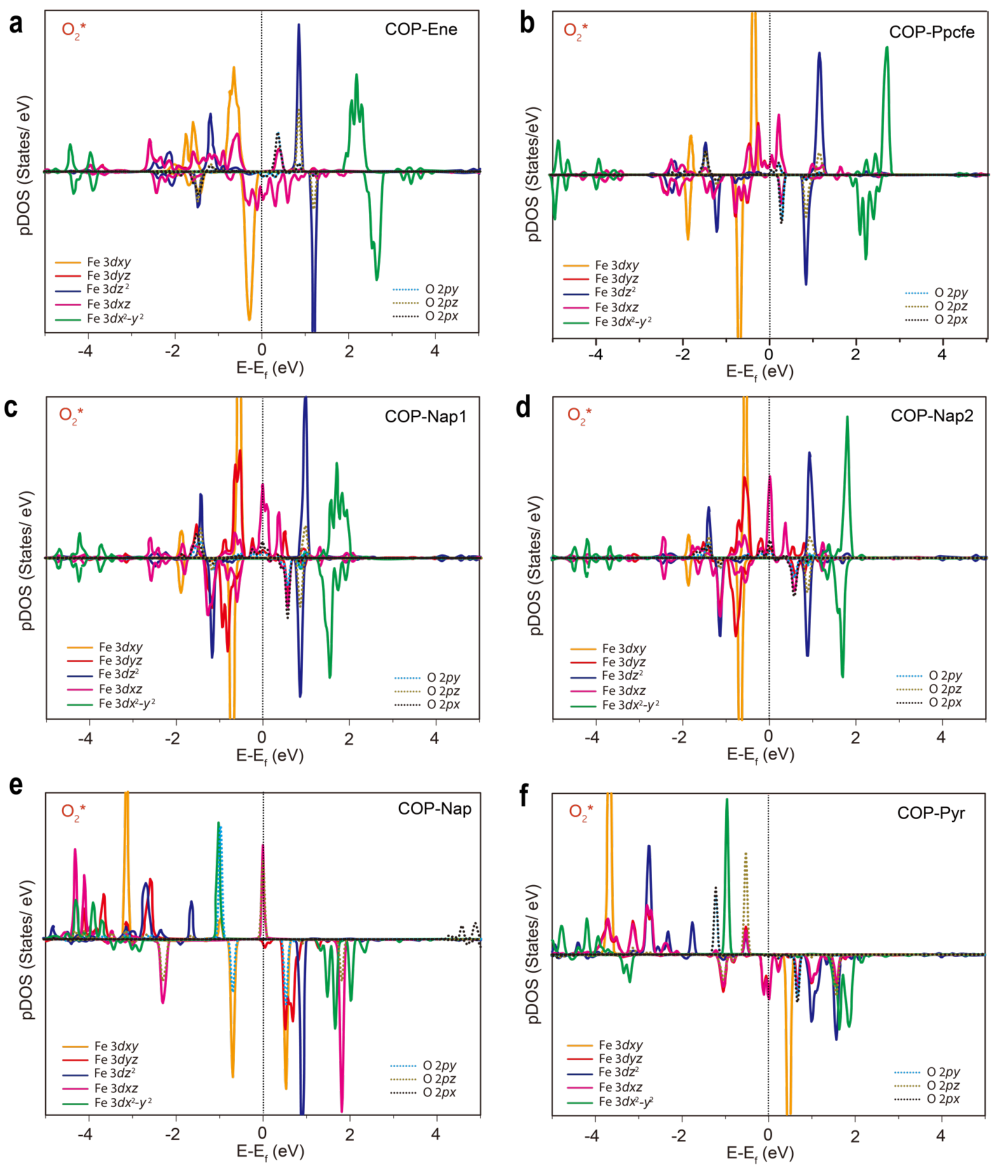

Supplementary Fig. 34. Projected density of states plots of 2*p* orbitals for O_2 and 3*d* orbitals for COP-Ene, COP-Ppcfe, COP-Nap1, COP-Nap2, COP-Nap and COP-Pyr.

Note: Supplementary Fig. 34. (the coordinates of $E\text{-}E_f$ are from -5 to 5 eV) is an entire picture of Fig. 6d in the text.

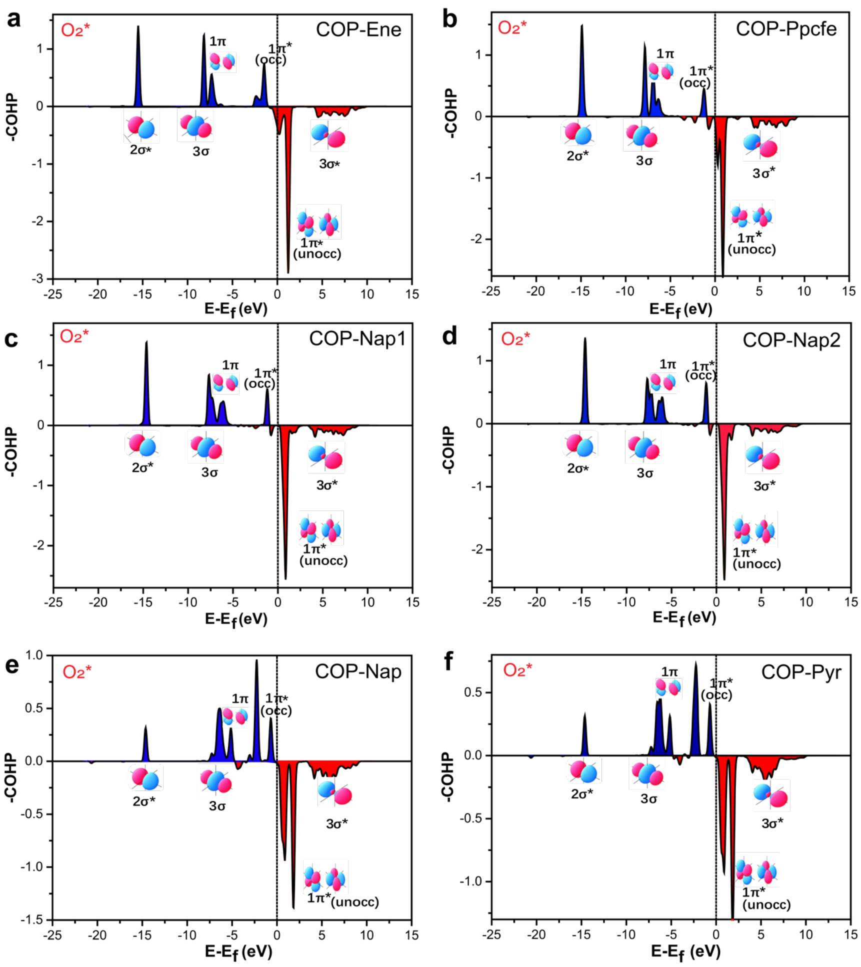

Supplementary Fig. 35. Crystal orbital Hamilton population (COHP) of O_2 absorbed on COP-Ene, COP-Ppcfe, COP-Nap1, COP-Nap2, COP-Nap and COP-Pyr surfaces.

As see from Supplementary Figs. 34-35, the unoccupied d orbitals of COPs accept electrons from the 3σ and 1π molecular orbitals of O_2, forming the bonding states to strengthen the O_2 adsorption. At the same time, the occupied d-orbitals of Fe atom in COPs back-donate electrons to the $1\pi^*$ orbital of O_2, leading to the partially occupied $1\pi^*$ orbital near the Fermi level. For FeN_4 moieties with abundant DDE, such as COP-Nap and COP-Pyr samples, electrons mainly transfer from $3d_{xz}$ and $3d_{yz}$ orbitals in Fe atom to $1\pi^*$ orbitals in dioxygen through π-back bonding. In contrast, for other COPs, superoxide species form when O_2 molecules are absorbed on Fe atom by end-on interaction, where electrons mainly transfer from the Fe $3d_{z^2}$ orbitals to the oxygen $1\pi^*$ orbitals and form σ bonds.

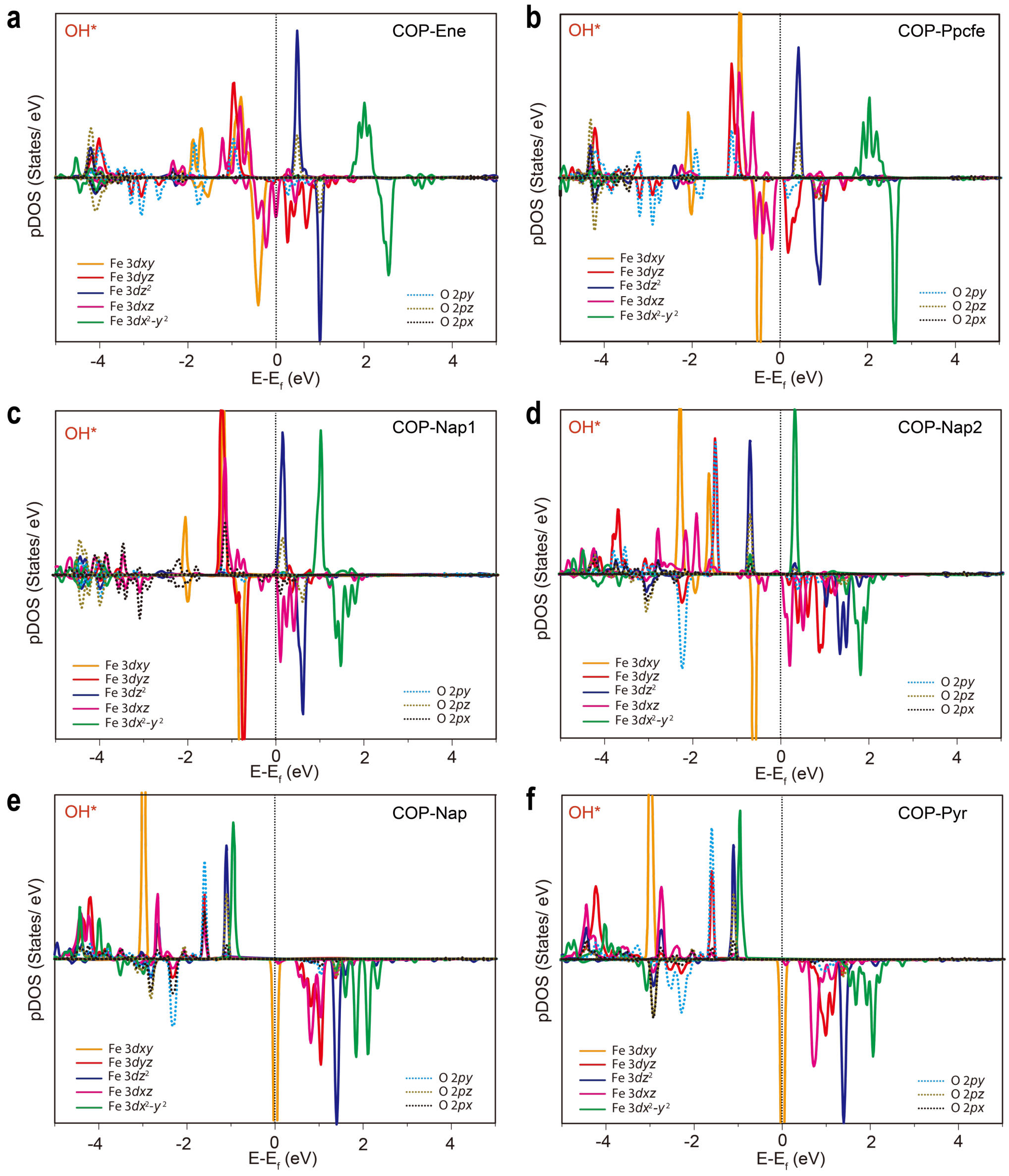

Supplementary Fig. 36. Projected density of states plots of 2*p* orbitals for OH* and 3*d* orbitals for COP-Ene, COP-Ppcfe, COP-Nap1, COP-Nap2, COP-Nap and COP-Pyr.

From the pDOS plots of 2*p* orbitals for OH* and 3*d* orbitals for six COP models, we can see that the 1π valence orbital in lone pair O $2p_x$, $2p_y$ electrons, and 3σ orbital in H 1*s* - O $2p_z$ of OH* intermediate occurred renormalizing when OH* reacted on 3*d* orbitals of Fe atom.

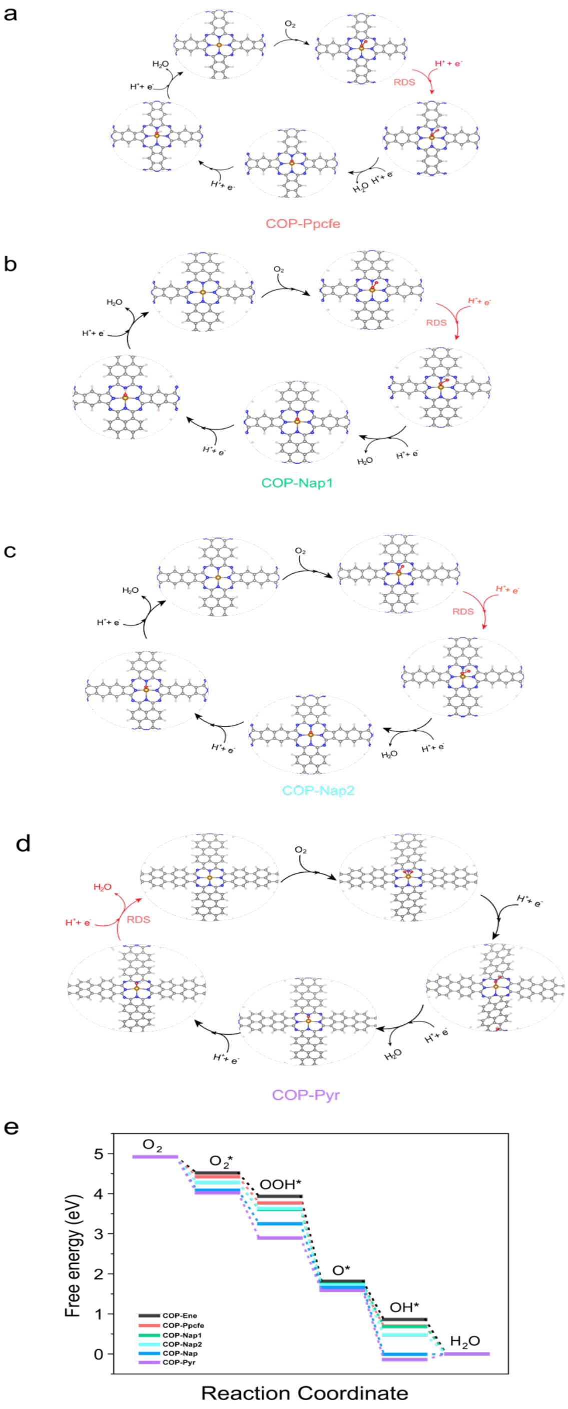

Supplementary Fig. 37. (a-d) Proposed ORR reaction scheme with the intermediates towards (a) COP-Ppcfe, (b) COP-Nap1, (c) COP-Nap2, (d) COP-Pyr. RDS is rate-determining step. The bule, grey, red, pink, and golden spheres represent N, C, O, H and Fe, respectively. (e) Free energy diagram for ORR on six configurations (COP-Ene, COP-Ppcfe, COP-Nap1, COP-Nap2, COP-Nap and COP-Pyr) at zero electrode potential.

As the degree of carbon conjugation increases, the potential-limiting step of adsorption energy gradually converts from oxygen activation to OH* desorption. It is worth noting that when the degree of delocalization of the carbon skeleton increases to a certain level (COP-Ppcfe or COP-Nap1), the overpotentials of the above two steps (O_2* +H^+ + e^-→ OOH*; OH* + H^++ e^-→ H_2O) at the equilibrium potential are nearly equal, and the entire four-electron reaction is under an optimal state.

Supplementary Tables:

Supplementary Table 1. EXAFS fitting parameters at the Fe K-edge various samples (S_0^2=0.82).

Sample	Path	C.N.	R (Å)	$\sigma^2\times10^3$ (Å^2)	ΔE (eV)	R factor
**Fe foil**	Fe-Fe	8*	2.47±0.01	4.5±1.2	-2.8±1.6	0.002
	Fe-Fe	6*	2.84±0.01	5.3±2.2	-5.2±3.4	
**FePc**	Fe-N	3.9±1.0	1.94±0.02	9.1±2.4	-0.4±3.6	0.008
	Fe-C	3.6±2.1	2.96±0.02	5.0±4.9	4.0±4.4	
**COP-Ene**	Fe-N	3.1±1.0	1.92±0.04	21.1±7.7	11.0±4.8	0.006
	Fe-C	5.2±1.0	3.20±0.02	5.2±3.4	7.2±1.8	
**COP-Ppcfe**	Fe-N	5.4±1.0	1.93±0.01	3.8±1.2	5.7±2.8	0.016
	Fe-C	7.6±2.7	2.98±0.02	2.2±2.3	9.8±3.4	
**COP-Nap**	Fe-N	3.8±0.5	1.98±0.02	12.0±3.7	1.2±3.1	0.014
	Fe-C	3.9±0.5	3.11±0.03	1.4±5.0	21.5±2.8	
**COP-Pyr**	Fe-N	3.7±0.3	1.98±0.01	8.8±1.9	3.1±1.6	0.017
	Fe-C	6.3±0.2	3.13±0.05	14.3±7.3	13.5±4.9	

$^a N$: coordination numbers; $^b R$: bond distance; $^c\sigma^2$: Debye-Waller factors; ^{d} ΔE_0: the inner potential correction. *R* factor: goodness of fit. * the experimental EXAFS fit of metal foil by fixing CN as the known crystallographic value.

Supplementary Table 2. The metal iron contents obtained by ICP-OES in four samples.

Sample	iron contents (wt.%)
**COP-Ene**	3.78
**COP-Ppcfe**	2.41
**COP-Nap**	1.93
**COP-Pyr**	1.30

Supplementary Table 3. Parameters derived from the fittings of Mössbauer spectra.

	Component	IS mm s^{-1}	QS mm s^{-1}	LW mm s^{-1}	Area %	Assignment
FePc	A1	0.19	0.42	0.46	15.33%	MS or LS FeN_4-O_2
	A2	0.18	2.72	0.29	69.43%	FeN_4
	A3	0.03	1.55	0.58	15.24%	HS FeN_4-O_2
COP-Ene	B1	0.18	0.54	0.56	92.76%	FeN_4
	B2	0.33	1.09	0.27	7.24%	HS FeN_4-O_2
COP-Ppcfe	C1	0.26	0.79	0.58	20.99%	MS or LS FeN_4-O_2
	C2	0.18	2.89	0.51	61.85%	FeN_4
	C3	0.09	1.89	0.57	17.16%	HS FeN_4-O_2
COP-Nap	D1	0.29	0.55	0.58	74.42%	FeN_4
	D2	0.26	1.74	0.59	25.58%	HS FeN_4-O_2
COP-Pyr	E1	0.33	0.56	0.58	81.94%	FeN_4
	E2	0.30	1.74	0.59	18.06%	HS FeN_4-O_2

Supplementary Table 4. Net charge for single Fe atom and the numbers of electrons gained for FeN_4 moiety on different models.

	**COP-Ene**	**COP-Ppcfe**	**COP-Nap1**	**COP-Nap2**	**COP-Nap**	**COP-Pyr**
**Net charge for single Fe atom (|e|)**	1.1228	1.1664	1.1243	1.1048	1.1512	1.1027
**The number of electrons gained for FeN_4 moiety (|e|)**	3.2766	3.4339	3.4971	3.5288	3.5553	3.6233

Supplementary Table 5. Total energies, zero-point energy corrections and entropic contributions to the free energy.

Species	E	ZPE	TS
**H_2O (0.035 bar)**	-14.218	0.56	0.67
**H_2**	-6.764	0.270	0.40
**O***	-	0.082	0.05
**OH***	-	0.385	0.07
**OOH***	-	0.457	0.16

Supplementary Table 6. Adsorption free energy (eV vs RHE) of OH, O, OOH, O_2 on different models.

	$\Delta G_{OH}*$	$\Delta G_{O}*$	$\Delta G_{OOH}*$	$\Delta G_{O2}*$
**COP-Ene**	0.8611	1.8154	3.9344	4.5180
**COP-Ppcfe**	0.6923	1.6586	3.7707	4.4218
**COP-Nap1**	0.6833	1.7445	3.6098	4.2789
**COP-Nap2**	0.4749	1.7222	3.6339	4.2807
**COP-Nap**	-0.0086	1.6586	3.2496	4.0871
**COP-Pyr**	-0.1419	1.5859	2.8954	4.0222

Supplementary Table 7. Reaction free energy (eV vs RHE) of elementary step for oxygen reduction at U_{RHE} = 0 V on different models.

	**ΔG_1**	**ΔG_2**	**ΔG_3**	**ΔG_4**	**ΔG_5**	**U^{onset} Potential**
**COP-Ene**	-0.4020	-0.5836	-2.1190	-0.9543	-0.8611	0.5836
**COP-Ppcfe**	-0.4982	-0.6511	-2.1121	-0.9663	-0.6923	0.6511
**COP-Nap1**	-0.6411	-0.6691	-1.8653	-1.0611	-0.6833	0.6691
**COP-Nap2**	-0.6393	-0.6468	-1.9118	-1.2472	-0.4749	0.4749
**COP-Nap**	-0.8329	-0.8374	-1.5910	-1.6673	0.0086	-0.0086
**COP-Pyr**	-0.8978	-1.1268	-1.3095	-1.7278	0.1419	-0.1419

Supplementary Table 8. Comparison of the acid ORR performance between COP-Ppcfe and other pyrolysis-free catalysts in literature.

**Catalyst**	**Electrolyte**	**Onset potential (mV)**	**Half-wave potential (mV vs RHE)**	**The number of electrons transferred**	**Loading (mg cm⁻²)**	**Reference**
COP-Ppcfe	0.1M $HClO_4$	877 vs RHE	748 vs RHE	3.87	0.255	**This work**
bi-FePc/GNS	0.5 M H_2SO_4	120 vs. Hg/Hg_2SO_4	-100 vs. Hg/Hg_2SO_4	3.64	0.398	3
FePc/C	0.5 M H_2SO_4	770 vs RHE	600 vs RHE	3.9	0.18	4
(DFTPP)Fe-Im-CNTs	0.1M $HClO_4$	1100 vs RHE	880 vs RHE	~ 4	1	5
FePPc/C	0.5 M H_2SO_4	890 vs RHE	800 vs RHE	3.9	0.65	6
16(F)FePc-CNT	0.1 M H_2SO_4	810 vs RHE	650 vs RHE	3.45	0.1	7
16(Cl)FePc	0.1 M H_2SO_4	550 vs SCE	400 vs SCE	3.45	0.1	8
14-membered macrocyclic Fe complex	0.5 M H_2SO_4	890 vs RHE	800 vs RHE	\	0.04	9
($phen_2N_2$)FeCl	0.1M $HClO_4$	700vs RHE	580 vs RHE	3.99	2.5	10

The oxygen reduction electrocatalysts obtained by pyrolysis-free methods mainly focus on the modification towards porphyrins or phthalocyanines molecular catalysts. And as far as we know, pyrolysis-free electrocatalysts with acid performance are extremely limited. In this work, the acid performance of COP-Ppcfe sample is leading among the same type of catalysts. In addition, the COP-Ppcfe sample shows outstanding oxygen reduction performance, far exceeding the loaded carbon black molecule FePc (Ref. 5), which also reflects the successful polymerization of COP-Ppcfe sample.

Supplementary References

1. Wan, X. et al. Fe-N-C electrocatalyst with dense active sites and efficient mass transport for high-performance proton exchange membrane fuel cells. *Nat. Catal.* **2**, 259-268 (2019).
2. Fei, H. et al. General synthesis and definitive structural identification of MN4C4 single-atom catalysts with tunable electrocatalytic activities. *Nat. Catal.* **1**, 63-72 (2018).
3. Li, T. et al. Enhanced activity and stability of binuclear iron (III) phthalocyanine on graphene nanosheets for electrocatalytic oxygen reduction in acid. *J. Power Sources* **293**, 511-518 (2015).
4. Baranton, S., Coutanceau, C., Roux, C., Hahn, F. & Léger, J.M. Oxygen reduction reaction in acid medium at iron phthalocyanine dispersed on high surface area carbon substrate: tolerance to methanol, stability and kinetics. *J. Electroanal. Chem.* **577**, 223-234 (2005).
5. Wei, P.J., Yu, G.Q., Naruta, Y. & Liu, J.G. Covalent grafting of carbon nanotubes with a biomimetic heme model compound to enhance oxygen reduction reactions. *Angew. Chem. Int. Ed.* **53**, 6659-6663 (2014).
6. Wang, X. et al. Iron polyphthalocyanine sheathed multiwalled carbon nanotubes: A high-performance electrocatalyst for oxygen reduction reaction. *Nano Res.* **9**, 1497-1506 (2016).
7. Abarca, G. et al. In search of the most active MN_4 catalyst for the oxygen reduction reaction. The case of perfluorinated Fe phthalocyanine. *J. Mater. Chem. A* **7**, 24776-24783 (2019).
8. Venegas, R. et al. Biomimetic reduction of O2in an acid medium on iron phthalocyanines axially coordinated to pyridine anchored on carbon nanotubes. *J. Mater. Chem. A* **5**, 12054-12059 (2017).
9. Moriya, M. et al. Fourteen-Membered Macrocyclic Fe Complexes Inspired by FeN_4-Center-Embedded Graphene for Oxygen Reduction Catalysis. J. *Phys. Chem. C* **124**, 20730-20735 (2020).

10. Marshall-Roth, T. et al. A pyridinic Fe-N_4 macrocycle models the active sites in Fe/N-doped carbon electrocatalysts. *Nat. Commun*. **11**, 5283 (2020).
